# The existence of nonnegative solutions for a nonlinear fractional *q*-differential problem via a different numerical approach

**DOI:** 10.1186/s13660-021-02612-z

**Published:** 2021-04-23

**Authors:** Mohammad Esmael Samei, Ahmad Ahmadi, Sayyedeh Narges Hajiseyedazizi, Shashi Kant Mishra, Bhagwat Ram

**Affiliations:** 1 Department of Mathematics, Bu-Ali Sina University, Hamedan, 65178 Iran; 2 Department of Mathematics, Institute of Science, Banaras Hindu University, Varanasi, 221005 India; 3 DST-Centre for Interdisciplinary Mathematical Sciences, Institute of Science, Banaras Hindu University, Varanasi, 221005 India; 4 Department of Medical Research, China Medical University Hospital, China Medical University, Taichung, Taiwan

**Keywords:** 34A08, 34B16, 34B18, Three-point conditions, Nonnegative solutions, Caputo fractional *q*-derivative, Numerical results

## Abstract

This paper deals with the existence of nonnegative solutions for a class of boundary value problems of fractional *q*-differential equation ${}^{c}\mathcal{D}_{q}^{\sigma }[k](t) = w (t, k(t), {}^{c} \mathcal{D}_{q}^{\zeta }[k](t) )$ with three-point conditions for $t \in (0,1)$ on a time scale $\mathbb{T}_{t_{0}}= \{ t : t =t_{0}q^{n}\}\cup \{0\}$, where $n\in \mathbb{N}$, $t_{0} \in \mathbb{R}$, and $0< q<1$, based on the Leray–Schauder nonlinear alternative and Guo–Krasnoselskii theorem. Moreover, we discuss the existence of nonnegative solutions. Examples involving algorithms and illustrated graphs are presented to demonstrate the validity of our theoretical findings.

## Introduction

It is recognized that fractional calculus provides a meaningful generalization for the classical integration and differentiation to any order. They can describe many phenomena in various fields of science and engineering such as control, porous media, electro chemistry, HIV-immune system with memory, epidemic model for COVID-19, chaotic synchronization, dynamical networks, continuum mechanics, financial economics, impulsive phenomena, complex dynamic networks, and so on (for more details, see [1–7]). It should be noted that most of the papers and books on fractional calculus are devoted to the solvability of linear initial value fractional differential equation in terms of special functions.

The study of *q*-difference equations has gained intensive interest in the last years. It has been shown that these equations have numerous applications in diverse fields and thus have evolved into multidisciplinary subjects. On the other hand, quantum calculus is equivalent to traditional infinitesimal calculus without the notion of limits. Fractional *q*-calculus, initially proposed by Jackson [8], is regarded as the fractional analogue of *q*-calculus. Soon afterward, it is further promoted by Al-Salam and Agarwal [9, 10], where many outstanding theoretical results are given. Its emergence and development extended the application of interdisciplinary to be further and aroused widespread attention of the scholars; see [11–23] and references therein.

In 2012, Zhoujin et al. considered the fractional differential equation

$$ {}^{c}\mathcal{D}^{\sigma }[k](s) + w \bigl( s , k(s), {}^{c}\mathcal{ D}^{\zeta }[k](s) \bigr) $$

for $0< s < 1$ and $\sigma \in (3, 4)$ under the boundary conditions $k(0) = k'(0) = k''(0) = 0$ and $k(1) = k(\varsigma )$ for $0 < \varsigma <1$, where ${}^{c}\mathcal{D}^{\sigma }$ denotes the Caputo fractional derivative, $\zeta > 0$, and $\sigma - \zeta \geq 1$. The existence results are derived by means of Schauder’s fixed-point theorem. Then Liang and Zhang [24] studied the existence and uniqueness of positive solutions by properties of the Green function, the lower and upper solution method, and the fixed point theorem for the fractional equation ${}\mathcal{D}_{q}^{\sigma }[k](s) + w ( s , k(s)) = 0$ for $0 < s < 1$ under the boundary conditions $k(0) = k'(0) = 0$ and $k'(1) = \sum_{i=1}^{m - 2} \ell _{i} k'(\varsigma _{i})$, where $2 < \sigma \leq 3$, and ${}^{c}\mathcal{ D}_{q}^{\sigma }$ is the Riemann–Liouville fractional derivative. In 2015, Zhang et al. [25] through the spectral analysis and fixed point index theorem obtained the existence of positive solutions of the singular nonlinear fractional differential equation

$$ \mathcal{D}^{\sigma }k(s) = w \bigl(S, k(s), \mathcal{D}^{\zeta }k(s) \bigr) $$

for almost all $s \in (0,1)$ with integral boundary value conditions $\mathcal{D }_{t}^{\zeta }k(0) =0$ and $\mathcal{D }^{\zeta }k(1) = \int _{0}^{1} \mathcal{ D}^{\zeta }k(r) { \,\mathrm{d}} \mu (r)$ where $\sigma \in (1, 2]$, $\zeta \in (0, 1]$, $w(s, k , l)$ may be singular at both $t=0$, 1 and $k=l =0$, $\int _{0}^{1} k(r) {\,\mathrm{d}}\mu (r)$ denotes the Riemann–Stieltjes integral with signed measure, in which $\mu : [0,1] \to \mathbb{R}$ is a function of bounded variation. In 2016, Ahmad et al. [16] investigated the existence of solutions for a *q*-antiperiodic boundary value problem of fractional *q*-difference inclusions

$$ {}^{c}\mathcal{D}_{q}^{\alpha }[k] (t) \in F \bigl( t, k(t), \mathcal{D}_{q} [k](t), \mathcal{D}_{q}^{2} [k](t) \bigr) $$

for $t \in [0,1]$, $q \in (0,1)$, $2 < \alpha \leq 3$, $0 < \beta \leq 3$, and $k(0) + k(1) =0$, $\mathcal{D}_{q} k(0) + \mathcal{D}_{q} k(1) =0$, $\mathcal{D}_{q}^{2} k(0) + \mathcal{D}_{q}^{2} k(1) =0$, where ${}^{c}\mathcal{D}_{q}^{\alpha }$ is the Caputo fractional *q*-derivative of order *α*, and $F: [0,1] \times \mathbb{R} \times \mathbb{R} \times \mathbb{R} \to \mathcal{P}( \mathbb{R})$ is a multivalued map with $\mathcal{P}(\mathbb{R})$ the class of all subsets of $\mathbb{R}$.

In 2018, Guezane-Lakoud and Belakroum [26] considered the existence and uniqueness of nonnegative solutions of the boundary value problem for nonlinear fractional differential equation ${}^{c}\mathcal{D}_{0}^{\alpha }[z](t)= \phi (t, z(t), {}^{c} \mathcal{D}_{0}^{\beta }[z](t) )$ for $t \in (0,1)$ under the conditions $z(0) = z''(0)=0$ and $z'(\tau ) = \alpha z''(1)$, where $\phi : [0,1 ] \times \mathbb{R}^{2} \to \mathbb{R}$ is a given function, *α*, *β* in $(2, 3)$ and $(0,1)$, respectively, $0 < \eta < 1$, and ${}^{c}\mathcal{D}_{0}^{\beta }$ denotes the Caputo fractional derivative. In 2019, Ren and Zhai [27] discussed the existence of a unique solution and multiple positive solutions for the fractional *q*-differential equation $\mathcal{D}_{q}^{\alpha }[x](t) + w(t, x(t))=0$ for each $t \in [0,1]$ with nonlocal boundary conditions $x(0) = \mathcal{D}_{q}^{ \alpha -2} [x](0) =0 $ and

$$ \mathcal{D}_{q}^{ \alpha -1} [x](1) = \mu (x) + \int _{0}^{\eta }\phi (r) \mathcal{D}_{q}^{\beta } [x](t) {\,\mathrm{d}}_{q}r, $$

where $\mathcal{ D}_{q}^{ \alpha }$ is the standard Riemann–Liouville fractional *q*-derivative of order *α* such that $2 < \alpha \leq 3$ and $\alpha - 1-\beta >0$, $q \in (0,1)$, $\phi \in L^{1}[0,1]$ is nonnegative, $\mu [x]$ is a linear functional given by $\mu [x] = \int _{0}^{1} x(t) {\,\mathrm{d}}N(t)$ involving the Stieltjes integral with respect to a nondecreasing function $N: [0,1] \to \mathbb{R}$ such that $N(t)$ is right-continuous on $[0,1)$, left-continuous at $t=1$, $N(0)=0$, and ${\,\mathrm{d}}N$ is a positive Stieltjes measure. Rehman et al. [28] developed Haar wavelets operational matrices to approximate the solution of generalized Caputo–Katugampola fractional differential equations. They introduced the Green–Haar approach for a family of generalized fractional boundary value problems and compared the method with the classical Haar wavelets technique. The existence of solutions for the multiterm nonlinear fractional *q*-integro-differential ${}^{c}D_{q}^{\alpha } [u](t)$ equation in two modes and inclusions of order $\alpha \in (n -1, n]$, where the natural number $n\ge 5$, with nonseparated boundary and initial boundary conditions was considered in [29]. In [30] the investigation is centered around the quantum estimates by utilizing the quantum Hahn integral operator via the quantum shift operator. In [20] the *q*-fractional integral inequalities of Henry–Gronwall type are presented.

Inspired by all the works mentioned, in this research, we investigate the existence and uniqueness of nonnegative solutions of the nonlinear fractional *q*-differential equation

1 $$ {}^{c}\mathcal{D}_{q}^{\sigma }[k](t) + w \bigl(t, k(t), {}^{c} \mathcal{D}_{q}^{\zeta }[k](t) \bigr)=0 $$

under the boundary conditions $k(0)=k''(0)=0$ and $k'(r) = \lambda k''(1)$ for $t\in J:=(0,1)$ and $0 < q <1$, where $w : \overline{J}\times \mathbb{R}^{2} \to \mathbb{R}$ is a given function with $\overline{J}:=[0,1]$, $2 < \sigma <3$, $\zeta \in J$, $r \in J$,and $\lambda >0$, and ${}^{c}\mathcal{D}_{q}^{\sigma }$ denotes the Caputo fractional *q*-derivative.

The rest of the paper is organized as follows. In Sect. 2, we cite some definitions and lemmas needed in our proofs. Section 3 treats the existence and uniqueness of solutions by using the Banach contraction principle and Leray–Schauder nonlinear alternative. Also, Sect. 3 is devoted to prove the existence of nonnegative solutions with the help of the Guo–Krasnoselskii theorem. Finally, Sect. 4 contains some illustrative examples showing the validity and applicability of our results. The paper concludes with some interesting observations.

## Preliminaries and lemmas

In this section, we recall some basic notions and definitions, which are necessary for the next goals. This section is devoted to state some notations and essential preliminaries acting as necessary prerequisites for the results of the subsequent sections. Throughout this paper, we will apply the time-scale calculus notation [31].

In fact, we consider the fractional *q*-calculus on the specific time scale $\mathbb{T}= \mathbb{R}$, where $\mathbb{T}_{t_{0}} = \{0 \} \cup \{ t: t=t_{0}q^{n} \}$ for nonnegative integer *n*, $t_{0} \in \mathbb{R}$, and $q \in (0,1)$. Let $a \in \mathbb{R}$. Define $[a]_{q} = (1-q^{a}) /(1- q)$ [8]. The power function $(x-y)_{q}^{n}$ with $n \in \mathbb{N}_{0} $ is defined by $(x-y)_{q}^{(n)}= \prod_{k=0}^{n-1} (x - yq^{k})$ for $n\geq 1$ and $(x-y)_{q}^{(0)}=1$, where *x* and *y* are real numbers, and $\mathbb{N}_{0} := \{ 0\} \cup \mathbb{N}$ [11]. Also, for $\alpha \in \mathbb{R}$ and $a \neq 0$, we have

$$ (x - y)_{q}^{(\alpha )}= x^{\alpha }\prod _{k=0}^{\infty } \bigl(x-yq^{k} \bigr)/ \bigl(x - yq^{ \alpha + k} \bigr). $$

If $y=0$, then it is clear that $x^{(\alpha )}= x^{\alpha }$ [12] (Algorithm 1). The *q*-gamma function is given by $\Gamma _{q}(z) = (1-q)^{(z-1)} / (1-q)^{z -1}$, where $z \in \mathbb{R} \backslash \{0, -1, -2, \ldots \}$ [8]. Note that $\Gamma _{q} (z+1) = [z]_{q} \Gamma _{q} (z)$. Algorithm 2 shows a pseudocode description of the technique for estimating the *q*-gamma function of order *n*. The *q*-derivative of a function *f* is defined by

$$ \mathcal{D}_{q} [f](x) = \frac{f(x) - f(qx)}{(1- q)x} $$

and $\mathcal{D}_{q} [f](0) = \lim_{x \to 0} \mathcal{D}_{q} [f](x)$, which is shown in Algorithm 3 [11]. Furthermore, the higher-order *q*-derivative of a function *f* is defined by $\mathcal{D}_{q}^{n} [f](x) = \mathcal{D}_{q}[ \mathcal{D}_{q}^{ n-1} [f]](x)$ for $n \geq 1$, where $\mathcal{D}_{q}^{0} [f](x) = f(x)$ [11]. The *q*-integral of a function *f* is defined on $[0,b]$ by

$$ I_{q} f(x) = \int _{0}^{x} f(s) \,\mathrm{d}_{q} s = x(1- q) \sum_{ k=0}^{ \infty } q^{k} f \bigl(x q^{k} \bigr) $$

for $0 \leq x \leq b$, provided that the series absolutely converges [11]. If $x\in [0, T]$, then $\int _{x}^{T} f(r) \,\mathrm{d}_{q} r = I_{q} [f](T) - I_{q} [f](x)$, which is equal to

$$ (1- q) \sum_{k=0}^{\infty } q^{k} \bigl[ T f \bigl(T q^{k} \bigr) - x f \bigl(x q^{k} \bigr) \bigr] $$

whenever the series exists. The operator $I_{q}^{n}$ is given by $I_{q}^{0} [h](x) = h(x) $ and $I_{q}^{n} [h](x) = I_{q} [I_{q}^{n-1} [h]] (x)$ for $n \geq 1$ and $h \in C([0,T])$ [11]. It has been proved that $D_{q} [I_{q} [h]](x) = h(x)$ and $I_{q} [D_{q} [h]](x) = h(x) - h(0)$ whenever *h* is continuous at $x =0$ [11]. The fractional Riemann–Liouville-type *q*-integral of a function *h* on $J=(0,1)$ for $\alpha \geq 0$ is defined by $\mathcal{I}_{q}^{0} [h](t) = h(t) $ and

2 $$\begin{aligned} \mathcal{I}_{q}^{\alpha }[h](t)& = \frac{1}{\Gamma _{q}(\alpha )} \int _{0}^{t} (t- qs)^{(\alpha - 1)} h(s) \,\mathrm{d}_{q}s \\ & = t^{\alpha }(1-q)^{\alpha }\sum_{k=0}^{ \infty } q^{k} \frac{ \prod_{i=1}^{k - 1} (1-q^{\alpha +i } ) }{ \prod_{i=1}^{k - 1} (1 - q^{i +1} ) } h \bigl(t q^{k} \bigr) \end{aligned}$$

for $t \in J$ [15, 17]. We can use Algorithm 5 for calculating $\mathcal{I}_{q}^{\alpha }[h](t)$ according to Eq. (2). Also, the Caputo fractional *q*-derivative of a function *h* is defined by

3 $$\begin{aligned} {}^{c}\mathcal{D}_{q}^{\alpha }[h](t) & = \mathcal{I}_{q}^{ [\alpha ]-\alpha } \bigl[\mathcal{D}_{q}^{[\alpha ]} [h] \bigr] (t) \\ & = \frac{1}{ \Gamma _{q} ([\alpha ] -\alpha )} \int _{0}^{t} (t- qs)^{ ( [\alpha ] - \alpha - 1 )} \mathcal{D}_{q}^{[ \alpha ]} [h] (s) \,\mathrm{d}_{q}s \end{aligned}$$

for $t \in J$ and $\alpha >0$ [17]. It has been proved that $\mathcal{I}_{q}^{\beta } [\mathcal{I}_{q}^{\alpha } [h]] (x) = \mathcal{I}_{q}^{\alpha + \beta } [h] (x)$ and $\mathcal{D}_{q}^{\alpha } [\mathcal{I}_{q}^{\alpha } [h]] (x)= h(x)$ for $\alpha , \beta \geq 0$ [17]. Algorithm 5 gives a pseudocode for $\mathcal{I}_{q}^{\alpha }[h](x)$.

**Algorithm 1:**
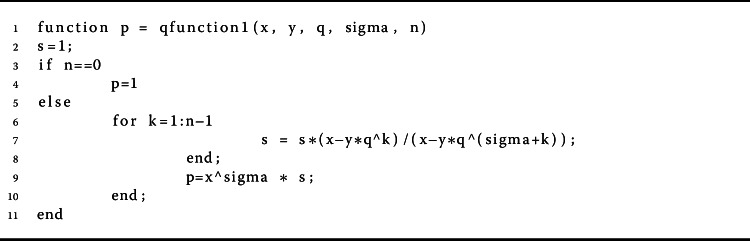
The proposed method for calculating $(x - y)_{q}^{(\alpha )}$

**Algorithm 2:**
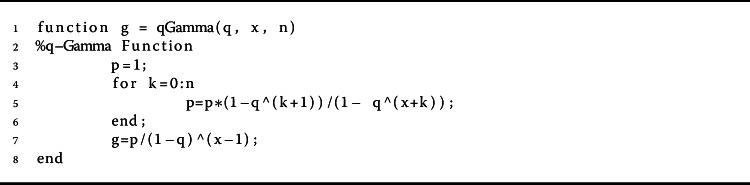
The proposed method for calculating $\Gamma _{q}(x)$

**Algorithm 3:**
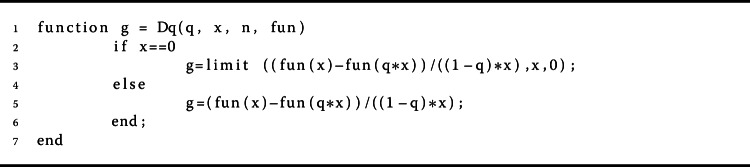
The proposed method for calculating $(D_{q} f)(x)$

### Lemma 2.1

([17])

*Let* *α*, $\beta \geq 0$ *and* $k \in L_{1} [a,b]$. *Then*

$$ \mathcal{I}_{q}^{\alpha } \bigl[\mathcal{ I}_{q}^{\beta }[k] \bigr](t) = \mathcal{ I}_{q}^{ \alpha +\beta } [k](t) = \mathcal{ I}_{q}^{\alpha } \bigl[\mathcal{I}_{q}^{\beta }[k] \bigr](t) $$

*and* ${}^{c}\mathcal{D}_{q}^{\alpha }[\mathcal{I}_{q}^{\beta }[k]](t) = [k](t)$ *for all* $t \in [a,b]$.

### Lemma 2.2

*Let* $\gamma > \lambda > 0 $. *Then* ${}^{c}\mathcal{D}_{q}^{\lambda }[ \mathcal{I}_{q}^{\gamma }[k]](t) = \mathcal{I}_{q}^{\gamma - \lambda } [k](t)$ *almost everywhere on* $t \in [a,b] $ *for* $k \in L_{1} [a,b] $, *and it is valid at any point* $x \in [a,b] $ *if* $k \in C [a,b] $.

### Lemma 2.3

([22])

*Let* $\sigma >0$ *and* $w \in L^{1}([a,b], \mathbb{R}^{+})$. *Then we have*

$$ \mathcal{ D}_{q}^{\sigma +1} [k](t) \leq \bigl\Vert \mathcal{ D}_{q}^{\sigma }[k] \bigr\Vert _{ L^{1}} $$

*for* $t \in [a,b]$.

To prove the theorems, we further apply the Leray–Schauder nonlinear alternative.

### Lemma 2.4

([32])

*Let* $\mathcal{A}$ *be a Banach space*, *let* $\mathcal{O}$ *be a bounded open subset of* $\mathcal{O}\in \mathcal{A}$, *and let* $\mathcal{H} : \overline{\mathcal{O}} \to \mathcal{A}$ *be a completely continuous operator*. *Then either there exist* $k \in \partial \mathcal{O}$ *and* $\lambda >1$ *such that* $\mathcal{H} (k) =\lambda k$, *or there exists a fixed point* $k^{*} \in \overline{\mathcal{O}}$.

### Theorem 2.5

*Let* $\mathfrak{B}$ *be a Banach space*, *and let* $\mathcal{C} \subset \mathfrak{B}$ *be a cone*. *Let* $\mathcal{O}_{1}$ *and* $\mathcal{O}_{2}$ *be open subsets of* $\mathfrak{B}$ *with* $0\in \mathcal{O}_{1}$, $\overline{\mathcal{O}}_{1} \subset \mathcal{O}_{2}$, *and let* $\Theta : \mathcal{C} \cap ( \overline{\mathcal{O}}_{2} \setminus \mathcal{O}_{1} ) \to \mathcal{C}$ *be a completely continuous operator such that*

(i) $\| \Theta (k)\| \leq \| k \|$ *for* $k\in \mathcal{C} \cap \partial \mathcal{O}_{1}$ *and* $\| \Theta (k)\| \geq \|k\|$ *for* $k \in \mathcal{C} \cap \partial \mathcal{O}_{2}$,
(ii) $\| \Theta (k)\| \geq \|k \|$ *for* $k \in \mathcal{C} \cap \partial \mathcal{O}_{1}$ *and* $\| \Theta (k)\| \leq \|k\|$ *for* $k\in \mathcal{C} \cap \partial \mathcal{O}_{2}$.

*Then* Θ *has a fixed point in* $\mathcal{C} \cap ( \overline{\mathcal{O}}_{2} \setminus \mathcal{O}_{1} )$.

## Main results

To facilitate exposition, we will provide our analysis in two separate folds. Now we give a solution of an auxiliary problem. Denote by $\mathcal{L}= L^{1} (\overline{J}, \mathbb{R})$ the Banach space of Lebesgue-integrable functions with the norm $\|k\|= \int _{0}^{1} |k(\xi )| {\,\mathrm{d}}\xi $.

### Lemma 3.1

*Let* $2 < \sigma < 3$ *and* $z \in C [a,b]$. *The unique solution of the* *q*-*fractional problem*

4 $$ \textstyle\begin{cases} {}^{c}\mathcal{D}_{q}^{\sigma }[k](t) = z (t), \\ k(0) = k''(0) =0, \qquad k'(r) = \lambda k''(1), \end{cases} $$

*for* $t \in J$ *is given by*

5 $$ k(t) = \frac{1}{ \Gamma _{q} (\sigma - 2)} \biggl[ \int _{0}^{1} {}_{1}G_{q}^{\zeta }(t , \xi ) z(\xi ) {\,\mathrm{d}}_{q}\xi - \frac{t}{ \sigma - 2} \int _{0}^{r} (r - q\xi )^{ (\sigma - 2)} z( \xi ) {\,\mathrm{d}}_{q}\xi \biggr], $$

*where*

6 $$ {}_{1}G_{q}^{\zeta }(t,\xi ) =\textstyle\begin{cases} \frac{(t - q\xi )^{(\sigma - 1)} }{(\sigma - 2)( \sigma - 1)} + \lambda t (1 - q\xi )^{( \sigma -3)}, & \xi \leq t, \\ \lambda t (1 - q\xi )^{( \sigma -3)},& \xi > t. \end{cases} $$

### Proof

First, by Lemma 2.1 and equation (4) we get

7 $$ k(t) =\mathcal{I}_{q}^{\sigma }[z](t) + d_{1} + d_{2} t + d_{3} t^{2}. $$

Differentiating both sides of (7) and using Lemma 2.2, we get

8 $$ \begin{aligned} &k'(t)= \mathcal{I}_{q}^{\sigma - 1} [z](t) + d_{2} + d_{3} t, \\ &k'' (t) = \mathcal{I}_{q}^{\sigma - 2}[z](t) + d_{3}. \end{aligned} $$

The first condition in equation (4) implies $d_{1} = d_{3} = 0$, and the second one gives

$$ d_{2} = \lambda \mathcal{I}_{q}^{ \sigma - 2} [z](1) - \mathcal{ I}_{q}^{ \sigma - 1} [z](r). $$

Substituting $d_{2}$ into equation (7), we obtain

9 $$ k(t) = \mathcal{I}_{q}^{\sigma }[z](t) + t \bigl[ \lambda \mathcal{I}_{q}^{ \sigma - 2} [z](1) - \mathcal{I}_{q}^{\sigma - 1} [z](r) \bigr], $$

which can be written as

10 $$\begin{aligned} k(t) ={}& \frac{1}{\Gamma _{q} (\sigma )} \int _{0}^{t} (t - q\xi )^{(\sigma -1) } z( \xi ) {\,\mathrm{d}}_{q}\xi \\ & {} + \frac{ \lambda t}{\Gamma _{q} (\sigma - 2) } \int _{0}^{1} (1 - q\xi )^{ (\sigma - 3)} z( \xi ) {\,\mathrm{d}}_{q}\xi \\ & {} - \frac{t}{\Gamma _{q} (\sigma - 1)} \int _{0}^{r} (\eta - q \xi )^{(\sigma -2) } z(\xi ) {\,\mathrm{d}}_{q}\xi . \end{aligned}$$

Indeed,

11 $$ k(t) = \frac{1}{\Gamma _{q} (\sigma - 2) } \biggl[ \int _{0}^{1} G(t, \xi ) z(\xi ) { \,\mathrm{d}}_{q}\xi - \frac{t}{\sigma - 2} \int _{0}^{r} (r - q\xi )^{(\sigma - 2)} z( \xi ) {\,\mathrm{d}}_{q}\xi \biggr], $$

where ${}_{1}G_{q}^{\zeta }(t, \xi )$ is defined by (6). The proof is complete. □

### Existence and uniqueness results

In this section, we prove the existence and uniqueness of nonnegative solutions in the Banach space $\mathfrak{B}$ of all functions $k \in C(\overline{J}) $ into $\mathbb{R} $ with the norm

$$ \Vert k \Vert = \max_{t\in \overline{J}} \bigl\vert k(t) \bigr\vert + \max_{t\in \overline{J}} \bigl\vert {}^{c}\mathcal{ D}_{q}^{\zeta }[k](t) \bigr\vert . $$

Note that ${}^{c}\mathcal{D}_{q}^{\zeta }k \in C (\overline{J})$ if $\zeta \in J$. Denote

$$ \mathcal{B} = \bigl\{ k \in \mathfrak{B} | k(t) \ge 0, t \in \overline{J} \bigr\} . $$

Throughout this section, we suppose that $w \in C(\overline{J} \times \mathbb{R}^{2}, \mathbb{R})$. We define the integral operator $\Theta : \mathfrak{B} \to \mathfrak{B}$ by

12 $$\begin{aligned} \Theta [k] (t)={}& \frac{1}{\Gamma _{q} (\sigma - 2)} \biggl[ \int _{0}^{1} {}_{1}G_{q}^{\zeta }(t, \xi ) w \bigl(\xi , k(\xi ), {}^{c} \mathcal{D}_{q}^{\sigma }[k]( \xi ) \bigr) {\,\mathrm{d}}_{q}\xi \\ & {} - \frac{t}{\sigma - 2} \int _{0}^{r} (r - q\xi )^{(\sigma - 2)} w \bigl(\xi , k(\xi ), {}^{c}\mathcal{D}_{q}^{\sigma }[k]( \xi ) \bigr) { \,\mathrm{d}}_{q}\xi \biggr]. \end{aligned}$$

Then we have the following lemma.

#### Lemma 3.2

*The function* $k \in \mathfrak{B}$ *is a solution of problem *(1) *if and only if* $\Theta [k](t) = k(t)$ *for* $t \in \overline{J}$.

#### Theorem 3.3

*The nonlinear fractional* *q*-*differential equation *(1) *has a unique solution* $k \in \mathfrak{B}$ *whenever there exist nonnegative functions* $g_{1}$, $g_{2} \in C(\overline{J}, \mathbb{R}^{+})$ *such that*

1. *for all* $k_{i}, l_{i} \in \mathbb{R}$ *with* $i=1, 2$ *and* $t \in \overline{J}$, *we have*
  13 $$ \bigl\vert w(t, k_{1}, k_{2})- w(t, l_{1}, l_{2}) \bigr\vert \leq \sum _{i=1}^{2} g_{i}(t) \vert k_{i} - l_{i} \vert , $$
2. $\Sigma _{A} = A_{1} + A_{2} < 1$ *and* $\Sigma _{B} = B_{1} + B_{2} < (1- \zeta ) \Gamma _{q} ( 1 - \zeta )$, *where*
  14 $$ \begin{aligned} A_{i} & = \bigl\Vert \mathcal{I}_{q}^{ \sigma -1} [g_{i}] \bigr\Vert _{L^{1}} + \vert \lambda \vert \mathcal{I}_{q}^{\sigma -2} [g_{i}] (1) + \mathcal{I}_{q}^{\sigma -1} [g_{i}] (r), \\ B_{i} & = \mathcal{I}_{q}^{ \sigma -1} \bigl( [g_{i}](1) + [g_{i}] (r) \bigr) + \vert \lambda \vert \mathcal{I}_{q}^{ \sigma -2} [g_{i}](1) \end{aligned} $$
  *for* $r \in J$ *and* $\lambda > 1$.

#### Proof

We transform the fractional *q*-differential equation to a fixed point problem. By Lemma 3.2 the fractional *q*-differential problem (1) has a solution if and only if the operator Θ has a fixed point in $\mathfrak{B}$. First, we will prove that Θ is a contraction. Let $k, l \in \mathfrak{B}$. Then

15 $$\begin{aligned} &\Theta [k] (t) - \Theta [l] (t) \\ &\quad = \frac{1}{\Gamma _{q} (\sigma - 2)} \biggl[ \int _{0}^{1} {}_{1}G_{q}^{\zeta }(t, \xi ) \bigl[ w \bigl( \xi , k(\xi ), {}^{c}\mathcal{D}_{q}^{\zeta }[k]( \xi ) \bigr) \\ & \qquad {}- w \bigl(\xi , l(\xi ), {}^{c}\mathcal{D}_{q}^{\zeta }[l]( \xi ) \bigr) \bigr] {\,\mathrm{d}}_{q}\xi \\ & \qquad{} - \frac{t}{\sigma - 2} \int _{0}^{r} (r - q\xi )^{(\sigma - 2)} \bigl[ w \bigl(\xi , k(\xi ), {}^{c}\mathcal{D}_{q}^{\zeta }[k]( \xi ) \bigr) \\ & \qquad {}- w \bigl( \xi , l(\xi ), {}^{c}\mathcal{D}_{q}^{\zeta }[l]( \xi ) \bigr) \bigr] {\,\mathrm{d}}_{q}\xi \biggr] \\ &\quad = \mathcal{I}_{q}^{\sigma } \bigl[ w \bigl( t , k(t), {}^{c} \mathcal{ D}_{q}^{\zeta }[k](t) \bigr) - w \bigl(t , l(t), {}^{c} \mathcal{D}_{q}^{\zeta }[l](t) \bigr) \bigr] \\ & \qquad{} + t \lambda \mathcal{I}_{q}^{ \sigma - 2} \bigl[ w \bigl(1, k(1), {}^{c}\mathcal{ D}_{q}^{\zeta }[k](1) \bigr) - w \bigl( 1 , l(1), {}^{c} \mathcal{ D}_{q}^{\zeta }[l](1) \bigr) \bigr] \\ & \qquad{} - t \mathcal{I}_{q}^{\sigma -2} \bigl[ w \bigl(r , k(r), {}^{c} \mathcal{D}_{q}^{\zeta }[k](r) \bigr) - w \bigl(r , l(r), {}^{c} \mathcal{D}_{q}^{\zeta }[l](r) \bigr) \bigr]. \end{aligned}$$

By inequality (13) we obtain

16 $$\begin{aligned} \Theta [k] (t) - \Theta [l] (t) \leq{}& \max _{t \in \overline{J}} \bigl\vert k (t) - l(t) \bigr\vert \\ & {} \times \bigl[ \mathcal{I}_{q}^{\sigma }[g_{1}](t) + \vert \lambda \vert \mathcal{I}_{q}^{\sigma -2} [g_{1}](1) + \mathcal{I}_{q}^{ \sigma -1} [g_{1}](r) \bigr] \\ & {} + \max_{ t\in \overline{J}} \bigl\vert {}^{c} \mathcal{D}_{q}^{\sigma }[k](t) - {}^{c} \mathcal{D}_{q}^{\sigma }[l](t) \bigr\vert \\ & {} \times \bigl[ \mathcal{I}_{q}^{\sigma }[g_{2}](t) + \vert \lambda \vert \mathcal{I}_{q}^{\sigma -2} [g_{2}](1) + \mathcal{ I}_{q}^{ \sigma -1} [g_{2}](r) \bigr]. \end{aligned}$$

On the other hand, Lemma 2.3 implies

17 $$\begin{aligned} \bigl\vert \Theta [k] (t) - \Theta [l] (t) \bigr\vert \leq {}&\bigl\Vert k(t)- l(t) \bigr\Vert \bigl[ \bigl\Vert \mathcal{I}_{q}^{ \sigma -1} [g_{1}] \vert + \vert \lambda \vert \mathcal{I}_{q}^{\sigma -2} [g_{1}](1) \\ & {} + \mathcal{I}_{q}^{\sigma -1} [g_{1}] (r)+ \bigl\| \mathcal{I}_{q}^{\sigma -1} [g_{2}] \bigr\Vert \\ & {} + |\lambda | \mathcal{I}_{q}^{\sigma -2} [g_{2}](1) + \mathcal{I}_{q}^{\sigma -1} [g_{2}](r) \bigr] \\ \leq {}& \bigl\Vert k(t) - l(t) \bigr\Vert (A_{1} + A_{2}). \end{aligned}$$

In view of (13), it yields

18 $$ \bigl\vert \Theta [k] (t) - \Theta [l] (t) \bigr\vert \leq \bigl\Vert k(t) - l(t) \bigr\Vert $$

for $t \in \overline{J}$. Also, we have

19 $$\begin{aligned} {}^{c}\mathcal{D}_{q}^{\zeta }[k](t) - {}^{c}\mathcal{D}_{q}^{\zeta }[l](t) ={}& \frac{1}{\Gamma _{q} (1 - \zeta ) } \int _{0}^{t} (t - q\xi )^{(- \zeta )} \\ & {} \times \bigl[ \bigl(\Theta [k] \bigr)' (\xi ) - \bigl( \Theta [l] \bigr)' (\xi ) \bigr] {\,\mathrm{d}}_{q} \xi , \end{aligned}$$

where

$$\begin{aligned} \bigl(\Theta [k] \bigr)'(t) ={}& \frac{1}{ \Gamma _{q}(\sigma -2)} \biggl[ \int _{0}^{1} {}_{1}H_{q}^{\zeta }(t, \xi ) w \bigl(\xi , k( \xi ), {}^{c}\mathcal{D}_{q}^{\zeta }[k]( \xi ) \bigr) \,\mathrm{d}_{q} \xi \\ & {} - \frac{1}{\Gamma _{q}( \sigma - 2)} \int _{0}^{r} (r - q\xi )^{ (\sigma - 2)} w \bigl(\xi , k(\xi ), {}^{c}\mathcal{D}_{q}^{\zeta }[k]( \xi ) \bigr) \mathrm{d }_{q} \xi \biggr], \end{aligned}$$

and

20 $$ {}_{1}H_{q}^{\zeta }(t, \xi ) = \frac{ \partial {}_{1}G_{q}^{\zeta }(t, \xi )}{\partial t} = \textstyle\begin{cases} \frac{ (t - \xi )^{(\sigma - 2)} }{ \sigma - 2} + \lambda (1 - q \xi )^{( \sigma -3)}, & \xi \leq t, \\ \lambda (1 - q \xi )^{( \sigma -3)},& \xi > t. \end{cases} $$

Therefore

21 $$\begin{aligned} &{}^{c}\mathcal{D}_{q}^{\zeta } \bigl[ \Theta [k] \bigr] (t) - {}^{c}\mathcal{D}_{q}^{\zeta } \bigl[\Theta [l] \bigr] (t) \\ &\quad = \frac{1}{ \Gamma _{q}(\sigma - 2 )\Gamma _{q}(1-\zeta )} \biggl[ \int _{0}^{t} (t-q \xi )^{(-\zeta )} \biggl( \int _{0}^{1} {}_{1}H_{q}^{\zeta }( \xi , qs) \\ & \qquad {}\times \bigl[ w \bigl(s, k(s), {}^{c}\mathcal{D}_{q}^{\zeta }[k](s) \bigr) - w \bigl(s, l(s), {}^{c}\mathcal{D}_{q}^{\zeta }[l](s) \bigr) \bigr] \,\mathrm{d}_{q}s - \frac{1}{ \Gamma _{q}( \sigma -2)} \int _{0}^{r} (r - qs)^{( \sigma -2)} \\ & \qquad {}\times \bigl[ w \bigl(s, k(s), {}^{c}\mathcal{D}_{q}^{\zeta }[k](s) \bigr)- w \bigl(s, l(s), {}^{c}\mathcal{ D}_{q}^{\zeta }[l](s) \bigr) \bigr] \,\mathrm{d}_{q}s \biggr) \,\mathrm{d}_{q} \xi \biggr]. \end{aligned}$$

Applying inequality (13), we get

22 $$\begin{aligned} &\bigl\vert {}^{c}\mathcal{D}_{q}^{\zeta } \bigl[\Theta [k] \bigr] (t)- {}^{c}\mathcal{D }_{q}^{\zeta } \bigl[\Theta [l] \bigr](t) \bigr\vert \\ &\quad \leq \frac{1}{ \Gamma _{q} (\sigma - 2) \Gamma _{q} (1 - \zeta )} \\ & \qquad{} \times \biggl[(t - q\xi )^{( -\zeta )} \int _{0}^{t} \biggl( \max_{ t\in \overline{J}} \bigl\vert k(t) - l(t) \bigr\vert \\ & \qquad{} \times \biggl( \int _{0}^{1} {}_{1}H_{q}^{\zeta }( \xi , s) g_{1}(s) {\,\mathrm{d}}_{q}s - \int _{0}^{r} \frac{(r - qs)^{(\sigma - 2)}}{ \sigma - 2} g_{1}(s) {\,\mathrm{d}}_{q}s \biggr) \\ & \qquad {}+ \max_{t \in \overline{J}} \bigl\vert {}^{c} \mathcal{ D}_{q}^{\zeta }[k](t) - {}^{c} \mathcal{D}_{q}^{\zeta }[l](t) \bigr\vert \\ & \qquad {}\times \biggl( \int _{0}^{1} {}_{1}H_{q}^{\zeta }( \xi ,s) g_{2}(s) {\,\mathrm{d}}_{q}s - \int _{0}^{r} \frac{(r - qs)^{(\sigma - 2)}}{\sigma - 2} g_{2}(s) {\,\mathrm{d}}_{q}s \biggr) \biggr) \biggr] {\,\mathrm{d}}_{q}\xi . \end{aligned}$$

Now let us estimate the term

$$ \int _{0}^{1} {}_{1}H_{q}^{\zeta }( \xi ,s) g_{1} (s) {\,\mathrm{d}}_{q}s - \int _{0}^{r} \frac{(r - qs)^{(\sigma - 2)} }{ \sigma - 2} g_{1}(s) {\,\mathrm{d}}_{q}s. $$

We have

23 $$\begin{aligned} &\biggl\vert \int _{0}^{1} {}_{1}G_{q}^{\zeta }( \xi , s) g_{1} (s) { \,\mathrm{d}}_{q}s - \frac{ \xi }{\sigma - 2} \int _{0}^{r} (r - qs)^{( \sigma -2)} g_{1}(s) {\,\mathrm{d}}_{q}s \biggr\vert \\ &\quad = \bigl\vert \Gamma _{q} (\sigma - 2) \bigl[ \mathcal{I}_{q}^{\sigma }[g_{1}] (\xi ) + \xi \bigl( \lambda \mathcal{I}_{q}^{\sigma -2} [g_{1}](1) + \mathcal{I}_{q}^{ \sigma -1} [g_{1}](r) \bigr) \bigr] \bigr\vert , \end{aligned}$$

24 $$\begin{aligned} &\biggl\vert \int _{0}^{1} {}_{1}H_{q}^{\zeta }( \xi ,s) g_{1}(s) {\,\mathrm{d}}_{q}s - \int _{0}^{r} \frac{(r - qs)^{(\sigma - 2)}}{\sigma - 2} g_{1}(s) {\,\mathrm{d}}_{q}s \biggr\vert \\ &\quad = \bigl\vert \Gamma _{q} (\sigma - 2) \bigl[ \mathcal{I}_{q}^{\sigma }[g_{1}]( \xi ) + \lambda \mathcal{I}_{q}^{ \sigma -2} [g_{1}](1) + \mathcal{I}_{q}^{ \sigma -1} [g_{1}](r) \bigr] \bigr\vert \\ & \quad \leq \Gamma _{q} (\sigma - 2) \bigl[ \mathcal{I}_{q}^{\sigma }[g_{1}] (\xi ) + \lambda \mathcal{ I}_{q}^{\sigma -2} [g_{1}](1) + \mathcal{I}_{q}^{\sigma -1} [g_{1}](r) \bigr] \\ & \quad \leq \Gamma _{q} (\sigma - 2) B_{1}, \end{aligned}$$

and, consequently, (22) becomes

$$\bigl\vert {}^{c}\mathcal{D}_{q}^{\zeta } \bigl[\Theta [k] \bigr](t) - {}^{c} \mathcal{D}_{q}^{\zeta } \bigl[\Theta [l] \bigr] (t) \bigr\vert = \frac{ \Vert k(t) - l(t) \Vert }{(1 - \zeta ) \Gamma _{q} (1 - \zeta ) } \Sigma _{B}. $$

By (15) this yields

25 $$ \bigl\vert {}^{c}\mathcal{D}_{q}^{\zeta } \bigl[\Theta [k] \bigr](t) - {}^{c} \mathcal{ D}_{q}^{\zeta } \bigl[\Theta [l] \bigr](t) \bigr\vert \leq \bigl\Vert k(t) - l(t) \bigr\Vert . $$

Taking into account (18)–(25), we obtain $\| \Theta [k] - \Theta [l] \| \leq \| k(t) - l(t) \|$ for $t \in \overline{J}$. From here the contraction principle ensures the uniqueness of solution for the fractional *q*-differential problem (1), which finishes the proof. □

We now give an existence result for the fractional *q*-differential problem (1).

#### Theorem 3.4

*Assume that* $w(0, 0, 0) \neq 0 $ *and there exist nonnegative functions* $g_{1}, g_{2}, g_{3} \in C(\overline{J}, \mathbb{R}^{+})$, *nondecreasing functions* $\phi _{1}, \phi _{2} \in C( \mathbb{R}^{+}, [0, \infty ])$, *and* $\eta > 0$ *such that*

26 $$ \bigl\vert w (t, k, \overline{k}) \bigr\vert \leq g_{1} (t) \phi _{1} \bigl( \bigl\vert k(t) \bigr\vert \bigr) + g_{2}(t) \phi _{2} \bigl( \bigl\vert \overline{k}(t) \bigr\vert \bigr) + g_{3}(t) $$

*for almost all* $(t, k, \overline{k}) \in \overline{J} \times \mathbb{R}^{2}$, *and*

27 $$ \bigl[ \phi _{1}(\eta ) + \phi _{2}( \eta ) +1 \bigr] \biggl( M_{1} + \frac{M_{2}}{(1 - \zeta ) \Gamma _{q} (1 - \zeta )} \biggr) < \eta , $$

*where *$M_{1} = \max_{t \in \overline{J}} \{ A_{1}, A_{2}, A_{3}\}$ *and* $M_{2} = \max_{t \in \overline{J}} \{ B_{1}, B_{2}, B_{3}\}$ *with* $A_{i}$ *and* $B_{i}$ *defined as in Theorem *3.3*by* (14). *Then the fractional* *q*-*differential problem *(1) *has at least one nontrivial solution* $k^{*} \in \mathfrak{B}$.

#### Proof

First, let us prove that Θ is completely continuous. It is clear that Θ is continuous since *w* and ${}_{1}G_{q}^{\zeta }$ are continuous. Let $\mathcal{B}_{\eta }= \{ k \in \mathfrak{B} : \|k\|\leq \eta \}$ be a bounded subset in $\mathfrak{B}$. We will prove that $\Theta ( \mathcal{B}_{\eta })$ is relatively compact.

(i) For $k \in \mathcal{B}_{\eta }$, using inequality (26), we get
  28 $$\begin{aligned} \bigl\vert \Theta [k](t) \bigr\vert \leq{}& \frac{1}{ \Gamma _{q}(\sigma -2)} \biggl[ \int _{0}^{1} \bigl\vert {}_{1}G_{q}^{\zeta }(t, \xi ) \bigr\vert \\ & {} \times \bigl[ g_{1}(\xi ) \phi _{1} \bigl( \bigl\vert k(\xi ) \bigr\vert \bigr) + g_{2} (\xi ) \phi _{2} \bigl( \bigl\vert {}^{c}\mathcal{D}_{q}^{\zeta }[k]( \xi ) \bigr\vert \bigr) + g_{3}(\xi ) \bigr] \,\mathrm{d}_{q}\xi \\ & {} + \frac{t}{ \Gamma _{q}( \sigma -2)} \int _{0}^{r} (r - q\xi )^{ (\sigma -2) } \\ & {} \times \bigl[ g_{1}(\xi ) \phi _{1} \bigl( \bigl\vert k(\xi ) \bigr\vert \bigr) + g_{2}( \xi ) \phi _{2} \bigl( \bigl\vert {}^{c}\mathcal{D}_{q}^{\zeta }[k]( \xi ) \bigr\vert \bigr) \bigr] \,\mathrm{d}_{q}\xi \biggr]. \end{aligned}$$
  Since $\phi _{1}$ and $\phi _{2}$ are nondecreasing, inequality (28) implies
  29 $$\begin{aligned} \bigl\vert \Theta [k](t) \bigr\vert \leq{}& \frac{1}{ \Gamma _{q}( \sigma -2)} \biggl[ \int _{0}^{1} \bigl\vert {}_{1}G_{q}^{\zeta }(t, \xi ) \bigr\vert \bigl[ g_{1}(\xi ) \phi _{1} \bigl( \Vert k \Vert \bigr) + g_{2}(\xi ) \phi _{2} \bigl( \Vert k \Vert \bigr) + g_{3}(\xi ) \bigr] \,\mathrm{d}_{q}\xi \\ & {} + \frac{t}{ \Gamma _{q} (\sigma - 2)} \int _{0}^{r} (r - q \xi )^{( \sigma - 2)} \bigl[ g_{1}(\xi ) \phi _{1} \bigl( \Vert k \Vert \bigr) + g_{2}(\xi ) \phi _{2} \bigl( \Vert k \Vert \bigr) \bigr] \,\mathrm{d}_{q}\xi \biggr] \\ \leq{} & \frac{1}{ \Gamma _{q} ( \sigma -2) } \biggl[ \int _{0}^{1} \bigl\vert {}_{1}G_{q}^{\zeta }(t, \xi ) \bigr\vert \bigl[ g_{1}(\xi ) \phi _{1} (\eta ) + g_{2}(\xi ) \phi _{2} (\eta ) + g_{3}(\xi ) \bigr] \,\mathrm{d}_{q} \xi \\ & {} + \frac{t}{ \Gamma _{q}( \sigma -2) } \int _{0}^{r} ( r - q \xi )^{ ( \sigma -2 )} \bigl[ g_{1}(\xi ) \phi _{1}(\eta ) + g_{2}(\xi ) \phi _{2}(\eta ) \bigr] \,\mathrm{d}_{q}\xi \biggr]. \end{aligned}$$
  Using similar techniques to get (18), this yields
  30 $$\begin{aligned} \bigl\vert \Theta [k](t) \bigr\vert < {}&\phi _{1}(\eta ) \bigl[ \bigl\Vert \mathcal{I}_{q}^{ \sigma -1} [g_{1}] \bigr\Vert _{L^{1}} + \vert \lambda \vert \mathcal{ I}_{q}^{ \sigma -2} [g_{1}](1) + \mathcal{ I}_{q}^{\sigma -1 } [g_{1}] (r) \bigr] \\ & {} + \phi _{2}(\eta ) \bigl[ \bigl\Vert \mathcal{I}_{q}^{ \sigma -1} [g_{2}] \bigr\Vert _{L^{1}} + \vert \lambda \vert \mathcal{I}_{q}^{ \sigma -2} [g_{2}](1) + \mathcal{I}_{q}^{\sigma -1} [g_{2}](r) \bigr] \\ &{} + \bigl\Vert \mathcal{I }_{q}^{ \sigma -1} [g_{3}] \bigr\Vert _{ L^{1}} + \vert \lambda \vert \mathcal{I}_{q}^{\sigma -2} [g_{3}] (1) + \mathcal{I}_{q}^{\sigma -1} [g_{3}] (r) \\ \leq {}& A_{1} \phi _{1}(\eta ) + A_{2} \phi _{2}(\eta ) + A_{3}. \end{aligned}$$
  Hence
  31 $$ \bigl\vert \Theta [k](t) \bigr\vert \leq M_{1} \bigl[ \phi _{1}(\eta ) + \phi _{2}(\eta ) + 1 \bigr]. $$
  Moreover, we have
  32 $$\begin{aligned} \bigl\vert \bigl( \Theta [k] \bigr)'(t) \bigr\vert ={}& \biggl\vert \frac{1}{ \Gamma _{q}(\sigma -2)} \biggl[ \int _{0}^{1} {}_{1}H_{q}^{\zeta }(t, \xi ) w \bigl(\xi , k(\xi ), {}^{c}\mathcal{D}_{q}^{\zeta }[k]( \xi ) \bigr) \,\mathrm{d}_{q}\xi \\ & {} - \frac{1}{(\sigma - 2)} \int _{0}^{r} (r - q\xi )^{ (\sigma -2)} w \bigl( \xi , k(\xi ), {}^{c}\mathcal{ D}_{q}^{\zeta }[k]( \xi ) \bigr) \,\mathrm{d}_{q}\xi \biggr] \biggr\vert \\ \leq {}&\frac{ 1 }{ \Gamma _{q}( \sigma -2)} \biggl\vert \int _{0}^{1} \bigl\vert {}_{1}H_{q}^{\zeta }(t, \xi ) \bigr\vert \bigl[ g_{1}(\xi ) \phi _{1}(\eta ) + g_{2}(\xi ) \phi _{2}(\eta ) + g_{3}(\xi ) \bigr] \,\mathrm{d}_{q}\xi \\ & {} - \frac{1}{(\sigma - 2)} \int _{0}^{r} (r - q\xi )^{(\sigma -2)} \bigl[ g_{1}(\xi ) \phi _{1}(\eta ) + g_{2}(\xi ) \phi _{2}(\eta ) \bigr] \,\mathrm{d}_{q}r \biggr\vert \\ \leq{}& \frac{1}{ \Gamma _{q}( \sigma - 2)} \biggl[ \biggl\vert \phi _{1}( \eta ) \int _{0}^{1} {}_{1}H_{q}^{\zeta }( t, \xi ) g_{1}(\xi ) \,\mathrm{d}_{q}\xi \\ & {} - \frac{1}{(\sigma - 2)} \int _{0}^{r} (r - q\xi )^{ (\sigma -2)} g_{1}(\xi ) \,\mathrm{d}_{q}\xi \biggr\vert \biggr] \\ & {} + \biggl[ \biggl\vert \phi _{2} (\eta ) \int _{0}^{1} {}_{1}H_{q}^{\zeta }(t, \xi ) g_{2}(\xi ) \,\mathrm{d}_{q}\xi \\ & {} - \frac{1}{(\sigma - 2)} \int _{0}^{r} (r - q\xi )^{(\sigma -2)} g_{2}(\xi ) \,\mathrm{d}_{q}\xi \biggr\vert \biggr] \\ &{} + \biggl[ \biggl\vert \int _{0}^{1} {}_{1}H_{q}^{\zeta }(t, \xi ) g_{3} (\xi ) \,\mathrm{d}_{q}\xi \\ & {} - \frac{1}{(\sigma - 2) } \int _{0}^{r} (r- q \xi )^{(\sigma -2)} g_{3} (\xi ) \,\mathrm{d}_{q}\xi \biggr\vert \biggr] \end{aligned}$$
  and
  33 $$ \begin{aligned} \bigl\vert \bigl( \Theta [k] \bigr)'(t) \bigr\vert & \leq B_{1} \phi _{1} (\eta ) + B_{2} \phi _{2}(\eta ) + B_{3}, \\ \bigl\vert \bigl(\Theta [k] \bigr)'(t) \bigr\vert & \leq M_{2} \bigl[ \phi _{1}(\eta ) + \phi _{2}( \eta ) + 1 \bigr]. \end{aligned} $$
  On the other hand, by (23) and (24) we obtain
  34 $$\begin{aligned} \bigl\vert {}^{c}\mathcal{D}_{q}^{\zeta } \bigl[\Theta [k] \bigr](t) \bigr\vert \leq{} & \frac{1}{\Gamma _{q}(1 - \zeta )} \\ & {} \times \int _{0}^{t} (t - q\xi )^{(-\zeta )} \bigl[ B_{1} \phi _{1}(\eta ) + B_{2} \phi _{2} (\eta ) + B_{3} \bigr] \,\mathrm{d}_{q} \xi \\ \leq{} & \frac{M_{2} }{ \Gamma _{q}(1 - \zeta ) } \int _{0}^{t} (t - q \xi )^{ (-\zeta )} \bigl[ \phi _{1}(\eta ) + \phi _{2}(\eta ) + 1 \bigr] \,\mathrm{d}_{q}\xi \\ \leq{} & \frac{M_{2}}{(1 - \zeta ) \Gamma _{q}(1 - \zeta )} \bigl[ \phi _{1}(\eta ) + \phi _{2}(\eta ) + 1 \bigr], \end{aligned}$$
  and from (31) and (32) we get
  35 $$ \bigl\Vert \Theta [k](t) \bigr\Vert = \bigl[ \phi _{1}(\eta ) + \phi _{2}(\eta ) + 1 \bigr] \biggl( M_{1} + \frac{M_{2}}{(1 - \zeta ) \Gamma _{q}(1 - \zeta )} \biggr). $$
  Then $\Theta (\mathcal{B}_{\eta })$ is uniformly bounded.
(ii) $\Theta ( \mathcal{ B}_{\eta })$ is equicontinuous. Indeed, for all $k \in \mathcal{B}_{\eta }$ and $t_{1}, t_{2} \in \overline{J}$ with $t_{1} < t_{2}$, denoting
  $$ M = \max_{t \in \overline{J}} \bigl\{ \bigl\vert w \bigl( t , k(t), {}^{c} \mathcal{ D}_{q}^{\zeta }[k](t) \bigr) \bigr\vert : \Vert k \Vert < \eta \bigr\} , $$
  we have
  36 $$\begin{aligned} \bigl\vert \Theta [k](t_{1}) - \Theta [k] (t_{2}) \bigr\vert &= \int _{t_{1}}^{t_{2}} \bigl\vert \bigl(\Theta [k] \bigr)'(\xi ) \bigr\vert \,\mathrm{d}_{q}\xi \\ & \leq \int _{t_{1}}^{t_{2}} \bigl[ B_{1} \phi _{1}(\eta ) + B_{2} \phi _{2}(\eta ) + B_{3} \bigr] \,\mathrm{d}_{q}\xi \\ & \leq (t_{1} - t_{2} ) \bigl[ B_{1} \phi _{1}(\eta ) + B_{2}\phi _{2}( \eta ) + B_{3} \bigr]. \end{aligned}$$
  Also, we have
  37 $$\begin{aligned} &\bigl\vert {}^{c}\mathcal{D}_{q}^{\zeta } \bigl[\Theta [k] \bigr] (t_{1}) - {}^{c} \mathcal{D}_{q}^{\zeta } \bigl[\Theta [l] \bigr]( t_{2}) \bigr\vert \\ &\quad = \biggl\vert \frac{1}{\Gamma _{q}(1 - \zeta )} \int _{0}^{t_{1}} (t_{1} - q\xi )^{(-\zeta )} \bigl( \Theta [k] \bigr)'(\xi ) \,\mathrm{d}_{q} \xi \\ &\qquad{} - \frac{1}{\Gamma _{q}(1 - \zeta )} \int _{0}^{t_{2}} (t_{2} - q \xi )^{ (-\zeta )} \bigl( \Theta [k] \bigr)'(\xi ) \,\mathrm{d}_{q} \xi \biggr\vert \\ & \quad \leq \frac{1}{\Gamma _{q}(1 - \zeta )} \int _{0}^{t_{1}} \bigl\vert (t_{1} - q\xi )^{(-\zeta )} - (t_{2} - qr)^{ (-\zeta )} \bigr\vert \bigl\vert \bigl( \Theta [k] \bigr)'(\xi ) \bigr\vert \,\mathrm{d}_{q}\xi \\ & \qquad{} + \frac{1}{\Gamma _{q}(1 - \zeta )} \int _{t_{1}}^{t_{2}} {(t_{2} - q\xi )^{(-\zeta )}} \bigl\vert \bigl( \Theta [k] \bigr)'(\xi ) \bigr\vert \,\mathrm{d}_{q}\xi . \end{aligned}$$
  Using (23), (24), and (32), this yields
  38 $$ \bigl\vert \bigl( \Theta [k] \bigr)'(t) \bigr\vert \leq M_{2} \bigl[\phi _{1} (\eta ) + \phi _{2}(\eta ) + 1 \bigr] $$
  and
  39 $$\begin{aligned} & \bigl\vert {}^{c}\mathcal{D}_{q}^{\zeta } \bigl[\Theta [k] \bigr](t_{1}) - {}^{c}\mathcal{ D}_{q}^{\zeta } \bigl[ \Theta [k] \bigr](t_{2}) \bigr\vert \\ &\quad \leq \frac{ M_{2} ( \phi _{1}(\eta ) + \phi _{2}( \eta ) + 1 ) }{ (1 - \zeta ) \Gamma _{q}(1 - \zeta )} \\ & \qquad{} \times \bigl[ 2( t_{2} - t_{1} )^{ ( 1 -\zeta )} + (t_{2})^{ (1- \zeta )} + (t_{1})^{ (1-\zeta )} \bigr]. \end{aligned}$$
  As $t_{1} \to t_{2}$ in (36) and (39), $|\Theta [k](t_{1}) - \Theta [k](t_{2})|$ and
  $$ \bigl\vert {}^{c}\mathcal{D}_{q}^{\zeta } \bigl[\Theta [k] \bigr](t_{1}) - {}^{c} \mathcal{ D}_{q}^{\zeta } \bigl[\Theta [l] \bigr](t_{2}) \bigr\vert $$
  tend to 0. Consequently, $\Theta (\mathcal{B}_{\eta })$ is equicontinuous.

By the Arzelá–Ascoli theorem we deduce that Θ is a completely continuous operator. Now we apply the Leray–Schauder nonlinear alternative to prove that Θ has at least one nontrivial solution in $\mathfrak{B}$. Letting $\mathcal{O} =\{ k \in \mathfrak{B} : \|k\| < \eta \}$, for any $k \in \partial \mathcal{O}$ such that $k = \tau \Theta [k](t)$, $0 < \tau < 1$, by (31) we get

40 $$ \bigl\vert k(t) \bigr\vert = \tau \bigl\vert \Theta [k](t) \bigr\vert \leq \bigl\vert \Theta [k](t) \bigr\vert \leq M_{1} \bigl(\phi _{1}(\eta ) + \phi _{2}( \eta ) + 1 \bigr). $$

Taking into account (34), we obtain

41 $$ \bigl\vert {}^{c}\mathcal{D}_{q}^{\zeta } \bigl[\Theta [k] \bigr] (t) \bigr\vert \leq \frac{M_{2}}{(1 - \zeta ) \Gamma _{q}(1 - \zeta )} \bigl[ \phi _{1}( \eta ) + \phi _{2}(\eta ) + 1 \bigr]. $$

From (40) and (41) we deduce that

42 $$ \Vert k \Vert \leq \bigl[ \phi _{1}(\eta ) + \phi _{2}(\eta ) + 1 \bigr] \biggl[ M_{1} + \frac{M_{2}}{(1 - \zeta ) \Gamma _{q}(1 - \zeta ) } \biggr]< \eta , $$

which contradicts the fact that $k \in \partial \mathcal{O}$. In this stage, Lemma 2.4 allows us to conclude that the operator Θ has a fixed point $k^{*}\in \mathcal{O}$, and thus the fractional *q*-differential problem (1) has a nontrivial solution $k^{*} \in \mathcal{O}$. The proof is completed. □

### Existence of nonnegative solutions

In this section, we investigate the positivity of nonnegative solutions for the fractional *q*-differential problem (1). To do this, we introduce the following assumptions.

1. $w(t, k, l) = \mu (t) \gamma (k, l)$, where $\mu \in C(\overline{J}, (0, \infty ) )$ and $\gamma \in C (\mathbb{R}^{+} \times \mathbb{R}, \mathbb{R}^{+})$.
2. $0 < \int _{0}^{1} (1- q \xi )^{ (\sigma -3)} \mu ( \xi ) \varrho _{q}( \xi ) \,\mathrm{d}_{q}\xi < \infty $, where
  $$ \varrho _{q}(\xi ) = \textstyle\begin{cases} \lambda ,& \xi > r, \\ \lambda - \frac{ (r - q\xi )^{(\sigma - 2) } (1 - q\xi )^{(\sigma -3)} }{ \sigma - 2}, & \xi \leq r. \end{cases} $$

Let us rewrite the function *k* as

$$ k(t) = \frac{1}{ \Gamma _{q} (\sigma - 2)} \int _{0}^{1} ( 1 - q \xi )^{ (\sigma -3)} {}_{2}G_{q}^{\zeta }( t,\xi ) \mu (\xi ) \gamma \bigl( k ( \xi ), {}^{c}\mathcal{D}_{q}^{\zeta }[k]( \xi ) \bigr) {\,\mathrm{d}}_{q} \xi , $$

where

43 $$ {}_{2}G_{q}^{\zeta }(t,\xi ) = \textstyle\begin{cases} \frac{(t - q \xi )^{(\sigma - 1)} (1- q \xi )^{ (\sigma - 3)} }{ (\sigma - 2)( \sigma - 1) } + \lambda t - \frac{t ( r - q\xi )^{(\sigma -2)} (1 - q \xi )^{(\sigma -3)}}{ \sigma -2}, & \xi \leq t, \xi \leq r, \\ \frac{(qt - q\xi )^{(\sigma - 1)} (1-q\xi )^{(\sigma -3)} }{(\sigma - 2)(\sigma - 1)} + \lambda t, & \xi \leq t, \xi > r, \\\lambda t - \frac{t (r - q\xi )^{(\sigma -2)} (1- q \xi )^{ (\sigma -3)}}{ \sigma -2},& \xi > t, \xi \leq r, \\ \lambda t, & \xi > t, \xi >r. \end{cases} $$

Hence

$$ {}^{c}\mathcal{D}_{q}^{\zeta }[k] (t)= \int _{0}^{1} (1 - q\xi )^{( \sigma -3)} {}_{2}H_{q}^{\zeta }(t,\xi ) \mu (\xi ) \gamma \bigl( k(\xi ), {}^{c}\mathcal{ D}_{q}^{\zeta }[k]( \xi ) \bigr) {\,\mathrm{d}}_{q}\xi , $$

where

44 $$ \begin{aligned}[b] &{}_{2}H_{q}^{\zeta } (t,q\xi ) \\ &\quad = \textstyle\begin{cases} \frac{(t - q\xi )^{(\sigma -\zeta - 1)} (1 - q\xi )^{(\sigma -3)} }{ \Gamma _{q}( \sigma -\zeta )} + \frac{\lambda t^{1 - \zeta } }{\Gamma _{q}(\zeta ) \Gamma _{q}( \sigma -2)}& \\ \quad - \frac{t^{1-\zeta }(r - q\xi )^{ (\sigma -2)} (1 - q\xi )^{ (\sigma -3)}}{\Gamma _{q}(\zeta ) \Gamma _{q}( \sigma -1)}, & \xi \leq t, \xi \leq r, \\ \frac{(t - q \xi )^{(\sigma -\zeta - 1)} ( 1 - q\xi )^{ (\sigma -3)} }{\Gamma _{q}(\sigma - \zeta )} + \frac{\lambda t^{ 1 - \zeta } }{ \Gamma _{q}(\zeta ) \Gamma _{q}( \sigma -2)}, & \xi \leq t, \xi > r, \\ \frac{\lambda t^{ 1 -\zeta } }{\Gamma _{q}(\zeta ) \Gamma _{q}( \sigma -2)} - \frac{ t^{1 - \zeta }(r-q\xi )^{ (\sigma - 2)} (1 - q\xi )^{ (\sigma -3)}}{ \Gamma _{q}(\zeta ) \Gamma _{q}(\sigma -1)},& \xi > t, \xi \leq r, \\ \frac{\lambda t^{ 1 - \zeta } }{ \Gamma _{q}(\zeta ) \Gamma _{q}( \sigma -2)}, & \xi > t, \xi >r. \end{cases}\displaystyle \end{aligned} $$

Now we give the properties of the Green function ${}_{2}H_{q}^{\zeta }(t, \xi )$.

#### Lemma 3.5

*If* $\lambda (\sigma - 2)\geq 1$, *then* ${}_{2}G_{q}^{\zeta }(t, \xi )$ *and* ${}_{2}H_{q}^{\zeta }(t,\xi ) $ *belong to* $C (\overline{J}^{2})$ *with* ${}_{2}G_{q}^{\zeta }(t, \xi )> 0$ *and* ${}_{2}H_{q}^{\zeta }(t,\xi ) >0$ *for all* $t, \xi \in \overline{J}$. *Furthermore*, *if* $t \in [\tau , 1]$, $\tau > 0$, *then for each* $\xi \in \overline{J}$, *we have*

$$ 0 < \tau \varrho _{q}(\xi ) \leq {}_{2}G_{q}^{\zeta }(t, \xi ) \le 2 \varrho _{q}(\xi ) $$

*and*

45 $$ 0 < \frac{\tau }{ \Gamma _{q}(\zeta ) \Gamma _{q}( \sigma -2)} \varrho _{q}(\xi ) \leq {}_{2}H_{q}^{\zeta }(t,\xi ) \leq \biggl[ \frac{1+ (\sigma -2) \Gamma _{q}( \zeta ) }{\Gamma _{q}(\zeta ) \Gamma _{q}( \sigma -2) } \biggr] \varrho _{q}(\xi ). $$

#### Proof

It is obvious that ${}_{2}G_{q}^{\zeta }(t,\xi ) \in C (\overline{J}^{2})$. Moreover, we have

$$\begin{aligned} &\lambda t - \frac{ t (r - q\xi )^{ (\sigma -2)} (1-\xi )^{(\sigma -3)} }{ \sigma - 2 } \\ &\quad = \frac{t}{\sigma -2} \bigl[ \lambda ( \sigma -2) - (1-q\xi )^{ ( \sigma - 3)} (r- q \xi )^{ (\sigma -2) } \bigr], \end{aligned}$$

which is positive if $\lambda (\sigma - 2) \geq 1$. Hence ${}_{2}G_{q}^{\zeta }(t,\xi )$ is nonnegative for all $t, \xi \in \overline{J}$. Let $t \in [ \tau , 1]$. It is easy to see that $\varrho _{q}(\xi ) \neq 0$. Then we have

$$\begin{aligned} {}_{2}G_{q}^{\zeta }(t,\xi )& = \frac{1}{ \lambda } \varrho _{q}(\xi ) \biggl[ t \lambda + \frac{(t - q\xi )^{(\sigma - 1)} (1-q\xi )^{(\sigma -3)} }{ (\sigma - 2 )(\sigma -1) } \biggr] \\ & \leq \frac{(1- q\xi )^{2} }{\sigma -1} + t \\ & \leq 2 \end{aligned}$$

whenever $e < \xi \leq t$,

$$\begin{aligned} {}_{2}G_{q}^{\zeta }(t,\xi ) ={}& \varrho _{q}(\xi ) \biggl[ \lambda - \frac{ ( r - q\xi )^{(\sigma -2)} ( 1 - q\xi )^{ (\sigma -3)}}{(\sigma -2)} \biggr]^{-1} \\ & {}\times \biggl[ \frac{ (t - q\xi )^{ (\sigma - 1)} (1 - q\xi )^{ (\sigma -3)} }{ ( \sigma -2)( \sigma -1)} + \lambda t - \frac{t ( r - q\xi )^{(\sigma -2)} (1 - q \xi )^{(\sigma -3)}}{ \sigma -2} \biggr] \\ = {}&t + \frac{(t - q\xi )^{ ( \sigma - 1)} (1 - q\xi )^{(\sigma -3) }}{ (\sigma - 1) [ \lambda (\sigma - 2) - ( r - q\xi )^{ (\sigma -2)} (1-q \xi )^{(\sigma - 3)} ]} \leq 2, \end{aligned}$$

whenever $\xi \leq t$, $\xi \leq r$,

$$\begin{aligned} {}_{2}G_{q}^{\zeta }(t,\xi )& = \varrho _{q}(\xi ) \biggl[ \lambda t ( 1-q \xi )^{(\sigma -3)} - \frac{t( e - q\xi )^{(\sigma -2)} }{ \sigma - 2} \biggr] \\ & \quad \times \biggl[ \lambda (1 - q\xi )^{(\sigma -3)} - \frac{ ( r - q\xi )^{ (\sigma -2) } }{ \sigma -2} \biggr]^{-1} \\ & = t\leq 2 \end{aligned}$$

whenever $t < \xi \leq r$, and ${}_{2}G_{q}^{\zeta }(t,\xi ) = t \varrho _{q}( \xi ) \leq 2$ whenever $t < \xi $, $e < \xi $. Thus

$$ {}_{2}G_{q}^{\zeta }(t,\xi ) \geq t \varrho _{q}(\xi ) \geq \tau \varrho _{q}(\xi ) $$

in all the cases. Since $\varrho _{q}(\xi )$ is nonnegative, we obtain

$$ 0< \tau \varrho _{q}(\xi )\leq {}_{2}G_{q}^{\zeta }( t,\xi ) \leq 2 \varrho _{q}(\xi ). $$

Similarly, we can prove that ${}_{2}H_{q}^{\zeta }( t,\xi )$ has the stated properties. The proof is completed. □

We recall the definition of a positive solution. A function *k* is called a positive solution of the fractional *q*-differential problem (1) if $k(t) \geq 0$ for all $t \in \overline{J}$.

#### Lemma 3.6

*If* $k \in \mathcal{B} $ *and* $\lambda (\sigma - 2) \geq 1$, *then the solution of the fractional* *q*-*differential problem *(1) *is nonnegative and satisfies*

46 $$ \min_{t \in [\tau , l]} \bigl( k(t) + {}^{c}\mathcal{D}_{q}^{\zeta }[k](t) \bigr) \geq \frac{ \tau ( 1+ \Gamma _{q}(\zeta )) }{ 1+ \sigma \Gamma _{q}(\zeta )} \Vert k \Vert . $$

#### Proof

First, let us remark that under the assumptions on *k* and *w*, the function ${}^{c}\mathcal{D}_{q}^{\zeta }[k]$ is nonnegative. Applying the right-hand side of inequality (45), we get

47 $$ k(t) \leq \frac{2}{\Gamma _{q}(\sigma - 2)} \int _{0}^{1} (1- q\xi )^{( \sigma -3)} \varrho (\xi ) \mu (\xi ) \gamma \bigl( k(\xi ), {}^{c} \mathcal{D}_{q}^{\zeta }[k](\xi ) \bigr) \,\mathrm{d}_{q}\xi . $$

Also, inequality (45) implies that

48 $$\begin{aligned} {}^{c}\mathcal{D}_{q}^{\zeta }[k] (t) & = \int _{0}^{1} (1 - q\xi )^{( \sigma -3)} {}_{2}H_{q}^{\zeta }(t,\xi ) \mu (\xi ) \gamma \bigl( k( \xi ), {}^{c}\mathcal{D }_{q}^{\zeta }[k]( \xi ) \bigr) \,\mathrm{d}_{q} \xi \\ & \leq \Lambda \int _{0}^{1} (1 - q\xi )^{(\sigma -3)} \mu (\xi ) \varrho (\xi ) \gamma \bigl( k(\xi ), {}^{c} \mathcal{D}_{q}^{\zeta }[k]( \xi ) \bigr) \,\mathrm{d}_{q}\xi , \end{aligned}$$

where $\Lambda = \frac{1+ (\sigma -2) \Gamma _{q}( \zeta ) }{\Gamma _{q}( \sigma -2) \Gamma _{q}(\zeta ) }$. Combining (47) and (48) yields

$$ \Vert k \Vert \leq \biggl[ \Lambda + \frac{2}{ \Gamma _{q}( \sigma - 2)} \biggr] \int _{0}^{1} (1- q\xi )^{(\sigma -3)} \mu ( \xi ) \varrho (\xi ) \gamma \bigl( k(\xi ), {}^{c} \mathcal{D}_{q}^{\zeta }[k]( \xi ) \bigr) \,\mathrm{d}_{q}\xi , $$

which is equivalent to

$$ \Vert k \Vert \leq \frac{1+ \sigma \Gamma _{q}(\zeta )}{ \Gamma _{q}(\zeta ) \Gamma _{q}( \sigma -2)} \int _{0}^{1} (1 - q\xi )^{(\sigma -3)} \mu (\xi ) \varrho (\xi ) \gamma \bigl( k(\xi ), {}^{c} \mathcal{D}_{q}^{\zeta }[k](\xi ) \bigr) \,\mathrm{d}_{q}\xi . $$

Indeed,

49 $$ \int _{0}^{1} (1-q\xi )^{(\sigma -3)} \mu ( \xi ) \varrho (\xi ) \gamma \bigl( k(\xi ), {}^{c} \mathcal{D}_{q}^{\zeta }[k](\xi ) \bigr) \,\mathrm{d}_{q}\xi \geq \frac{\Gamma _{q}(\zeta ) \Gamma _{q}( \sigma -2) }{1+ \sigma \Gamma _{q}(\zeta ) } \Vert k \Vert . $$

In view of the left-hand side of (45), we obtain that for all $t \in [\tau , l]$,

50 $$ k(t) \geq \frac{ \tau }{ \Gamma _{q}( \sigma -2)} \int _{0}^{1} (1 - q \xi )^{ (\sigma -3)} \mu (\xi ) \varrho (\xi ) \gamma \bigl( k(\xi ), {}^{c}\mathcal{ D}_{q}^{\zeta }[k](\xi ) \bigr) \,\mathrm{d}_{q} \xi . $$

On the other hand, we have

51 $$\begin{aligned} {}^{c}\mathcal{D}_{q}^{\zeta }[k](t) \geq{}& \frac{ \tau }{ \Gamma _{q}(\zeta ) \Gamma _{q}( \sigma -2)} \\ & {} \times \int _{0}^{1} (1-q\xi )^{( \sigma -3)} \mu ( \xi ) \varrho (\xi ) \gamma \bigl( k(\xi ), {}^{c} \mathcal{D}_{q}^{\zeta }[k]( \xi ) \bigr) \,\mathrm{d}_{q}\xi . \end{aligned}$$

From (50) and (51) we get

$$\begin{aligned} &\min_{t \in [\tau , l]} \bigl( k(t) + {}^{c} \mathcal{D}_{q}^{\zeta }[k](t) \bigr) \\ &\quad \geq \frac{ \tau ( 1 + \Gamma _{q} (\zeta ) )}{ \Gamma _{q}(\zeta ) \Gamma _{q} (\sigma - 2 )} \int _{0}^{1} (1 - q\xi )^{(\sigma -3)} \mu (\xi ) \varrho (\xi ) \gamma \bigl( k(\xi ), {}^{c} \mathcal{D}_{q}^{\zeta }[k]( \xi ) \bigr) \,\mathrm{d}_{q}\xi , \end{aligned}$$

and by (49) we deduce that

$$\begin{aligned} \min_{t \in [\tau , l]} \bigl( k(t) + {}^{c} \mathcal{D}_{q}^{\zeta }[k](t) \bigr) \geq \frac{\tau ( 1 + \Gamma _{q}(\zeta ) )}{\Gamma _{q}(\zeta ) \Gamma _{q}(\sigma -2)} \Vert k \Vert . \end{aligned}$$

This completes the proof. □

Define the quantities $L_{0}$ and $L_{\infty }$ by

$$\begin{aligned} L_{0} & = \lim_{( \vert k \vert + \vert l \vert ) \to 0} \frac{\gamma (k, l)}{ \vert k \vert + \vert l \vert }, \\ L_{\infty }& = \lim_{( \vert k \vert + \vert l \vert ) \to \infty } \frac{\gamma (k, l)}{ \vert k \vert + \vert l \vert }. \end{aligned}$$

The case of $L_{0}=0$ and $L_{\infty }= \infty $ is called the superlinear case, and the case of $L_{0} = \infty $ and $L_{\infty }= 0$ is called the sublinear case. To prove the main result of this section, we apply the well-known Guo–Krasnoselkii fixed point Theorem 2.5 on a cone.

#### Theorem 3.7

*Under the assumptions of Lemma *3.6, *the fractional* *q*-*differential problem *(1) *has at least one nonnegative solution in the both superlinear and sublinear cases*.

#### Proof

First, we define the cone

52 $$ \mathcal{C} = \biggl\{ k \in \mathcal{B} | \min _{t \in [ \tau , l]} \bigl( k(t) + {}^{c}\mathcal{D}_{q}^{\zeta }[k](t) \bigr) \geq \frac{\tau ( 1 + \Gamma _{q}(\zeta ) )}{ \Gamma _{q}(\zeta ) \Gamma _{q}(\sigma -2) } \Vert k \Vert \biggr\} . $$

We can easily check that $\mathcal{C}$ is a nonempty closed convex subset of $\mathfrak{B}$, and hence it is a cone. Using (3.6), we see that $\Theta [\mathcal{C}] \subset \mathcal{C}$. Also, from the proof of Theorem (3.4) we know that Θ is completely continuous in $\mathfrak{B}$. Let us prove the superlinear case.

1. Since $L_{0} = 0$, for any $\varepsilon > 0$, there exists $\delta _{1} > 0$ such that $\gamma (k, l) \leq \varepsilon (|k|+ |l|)$ for $0 < |k|+ |l| < \delta _{1}$. Letting $\mathcal{O}_{1} = \{ k \in \mathfrak{B} : \| k \| \leq \delta _{1} \}$, for any $k \in \mathcal{C} \cap \partial \mathcal{O}_{1} $, this yields
  53 $$\begin{aligned} \Theta [k](t) ={}& \frac{1}{\Gamma _{q}( \sigma -2)} \int _{0}^{1} (1- q \xi )^{(\sigma -3)} \\ & {} \times {}_{2}G_{q}^{\zeta }(t,\xi ) \mu (\xi ) \gamma \bigl( k( \xi ), {}^{c}\mathcal{D}_{q}^{\zeta }[k]( \xi ) \bigr) \,\mathrm{d}_{q} \xi \\ \leq {}&\frac{ 2 \varepsilon \Vert k \Vert }{ \Gamma _{q} ( \sigma - 2 )} \int _{0}^{1} (1 - q\xi )^{(\sigma -3)} \mu (\xi ) \varrho (\xi ) \,\mathrm{d}_{q} \xi . \end{aligned}$$
  Moreover, we have
  54 $$\begin{aligned} {}^{c}\mathcal{D}_{q}^{\zeta }[k](t) & \leq \Lambda \int _{0}^{1} (1 - q \xi )^{(\sigma -3)} \mu (\xi ) \varrho (r) \gamma \bigl( k(\xi ), {}^{c} \mathcal{D}_{q}^{\zeta }[k](r) \bigr) \,\mathrm{d}_{q} \xi \\ & \leq \Lambda \varepsilon \Vert k \Vert \int _{0}^{1} (1-q\xi )^{(\sigma -3)} \mu ( \xi ) \varrho (\xi ) \,\mathrm{d}_{q}\xi . \end{aligned}$$
  From (53) and (54) we conclude
  55 $$\begin{aligned} \begin{aligned} & \bigl\Vert \Theta [k] \bigr\Vert \leq \biggl[ \frac{2}{\Gamma _{q}( \sigma -2)} + \Lambda \biggr] \varepsilon \Vert k \Vert \int _{0}^{1} (1-q\xi )^{(\sigma -3)} \mu ( \xi ) \varrho (\xi ) \,\mathrm{d}_{q}\xi , \\ &\bigl\Vert \Theta [k] \bigr\Vert \leq \biggl[ \frac{ \sigma \Gamma _{q}(\zeta ) + 1 }{ \Gamma _{q}( \zeta ) \Gamma _{q}( \sigma - 2)} \biggr] \varepsilon \Vert k \Vert \int _{0}^{1} (1- q \xi )^{(\sigma -3)} \mu ( \xi ) \varrho (\xi ) \,\mathrm{d}_{q}\xi . \end{aligned} \end{aligned}$$
  In view of assumption (A2), we can choose *ε* such that
  56 $$ \biggl[ \int _{0}^{1} (1-q\xi )^{(\sigma -3)} \mu ( \xi ) \varrho (\xi ) \,\mathrm{d}_{q}\xi \biggr] \varepsilon \leq \frac{ \Gamma _{q}(\zeta ) \Gamma _{q}( \sigma -2) }{1+\sigma \Gamma _{q}( \zeta ) }. $$
  Inequalities (55) and (56) imply that $\|\Theta [ k](t)\| \leq \|k(t)\|$ for each $k \in \mathcal{C} \cap \partial \mathcal{O}_{1}$.
2. Second, in view of $L_{\infty }=0$, for any $M > 0$, there exists $\delta _{2}>0 $ such that $w_{1}(k, l) \geq M(|k|+ |l|)$ for $(|k|+ |l|) \geq \delta _{2}$. Take
  $$ \delta = \max \biggl\{ 2\delta _{1}, \frac{ 1+ \sigma \Gamma _{q}(\zeta ) }{\tau ( 1 + \Gamma _{q}(\zeta ) )} \delta _{2} \biggr\} $$
  and denote by $\mathcal{O}_{2}$ the open set $\{ k \in \mathfrak{B} : \|k\| \leq \delta \|$. If $k \in \mathcal{C} \cap \partial \mathcal{O}_{2}$, then
  $$\begin{aligned} \min_{t \in [\tau , l]} \bigl( k(t) + {}^{c} \mathcal{D}_{q}^{\zeta }[k](t) \bigr) & \geq \frac{\tau ( 1 + \Gamma _{q}(\zeta ))}{ 1 +\sigma \Gamma _{q}(\zeta )} \Vert k \Vert \\ & = \frac{ \tau ( 1 + \Gamma _{q}(\zeta ) )}{1+ \sigma \Gamma _{q}(\zeta ) } \delta \geq \delta _{2}. \end{aligned}$$
  Using the left-hand side of (45) and Lemma (3.6), we obtain
  57 $$\begin{aligned} \Theta [k](t)\geq {}&\frac{\tau }{ \Gamma _{q}( \sigma -2)} \int _{0}^{1} (1-q\xi )^{(\sigma -3)} \\ & {} \times \mu (\xi ) \varrho (\xi ) \gamma \bigl( k(\xi ), {}^{c} \mathcal{D}_{q}^{\zeta }[k](\xi ) \bigr) \,\mathrm{d}_{q}\xi \\ \geq{}& \frac{\tau M }{ \Gamma _{q} (\sigma - 2 )} \Vert k \Vert \int _{0}^{1} (1- q \xi )^{(\sigma -3)} \mu (\xi ) \varrho (\xi ) \,\mathrm{d}_{q} \xi . \end{aligned}$$
  Moreover, by inequality (51) we get
  58 $$\begin{aligned} {}^{c}\mathcal{D}_{q}^{\zeta } \bigl[ \Theta [k] \bigr](t) \geq{} & \frac{\tau }{ \Gamma _{q}(\zeta ) \Gamma _{q}(\sigma -2)} \\ & {} \times \int _{0}^{1} (1 - q\xi )^{ (\sigma -3)} \mu (\xi ) \varrho (\xi ) \gamma \bigl( k(\xi ), {}^{c} \mathcal{D}_{q}^{\zeta }[k]( \xi ) \bigr) \,\mathrm{d}_{q}\xi \\ \geq{} & \frac{\tau }{\Gamma _{q}(\zeta ) \Gamma _{q}(\sigma -2)} M \Vert k \Vert \int _{0}^{1} (1-q\xi )^{(\sigma -3)} \mu (r) \varrho (\xi ) \,\mathrm{d}_{q}\xi . \end{aligned}$$
  In view of inequalities (57) and (58), we can write
  $$\begin{aligned} &\Theta [k](t) + {}^{c}\mathcal{D}_{q}^{\zeta } \bigl[\Theta [k] \bigr](t) \\ & \quad \geq \frac{\tau ( 1 + \Gamma _{q}(\zeta )) }{\Gamma _{q}(\zeta ) \Gamma _{q}(\sigma -2)} M \Vert k \Vert \int _{0}^{1} (1-q\xi )^{(\sigma -3)} \mu ( \xi ) \varrho ( \xi ) \,\mathrm{d}_{q}\xi . \end{aligned}$$
  Let us choose *M* such that
  $$ \Gamma _{q}(\zeta ) \Gamma _{q}(\sigma -2) \leq M \biggl[ \tau \bigl( 1 + \Gamma _{q}(\zeta ) \bigr) \int _{0}^{1} (1-q\xi )^{(\sigma -3)} \mu ( \xi ) \varrho (\xi ) \,\mathrm{d}_{q}\xi \biggr]. $$
  Then we get $\Theta [k](t) + {}^{c}\mathcal{ D}_{q}^{\zeta }[ \Theta [k]](t) \geq \| k \|$. So, $\| \Theta [k](t)\| \geq \| k(t) \|$ for each $k \in \mathcal{C} \cap \partial \mathcal{O}_{2}$.

The first part of Theorem (2.5) implies that Θ has a fixed point in $\mathcal{C} \cap ( \overline{ \mathcal{O}}_{2} \setminus \mathcal{O}_{1} )$ such that $\delta _{2} \leq \|k\| \leq \delta $. To prove the sublinear case, we apply similar techniques. The proof is complete. □

## Some illustrative examples

Herein, we give some examples to show the validity of the main results. In this way, we give a computational technique for checking problem (1). We need to present a simplified analysis that is able to execute the values of the *q*-gamma function. For this purpose, we provided a pseudocode description of the method for calculation of the *q*-gamma function of order *n* in Algorithms 2, 3, 4, and 5; for more detail, follow these address https://www.dm.uniba.it/members/garrappa/software.

**Algorithm 4:**
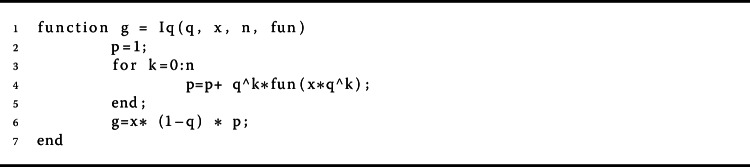
The proposed method for calculating $\int _{a}^{b} f(r) d_{q} r$

**Algorithm 5:**
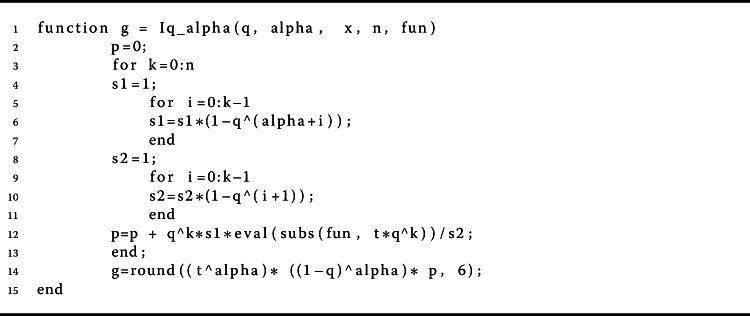
The proposed method for calculating $I_{q}^{\alpha}[x]$

For problems for which the analytical solution is not known, we will use, as reference solution, the numerical approximation obtained with a tiny step *h* by the implicit trapezoidal PI rule, which, as we will see, usually shows an excellent accuracy [33]. All the experiments are carried out in MATLAB Ver. 8.5.0.197613 (R2015a) on a computer equipped with a CPU AMD Athlon(tm) II X2 245 at 2.90 GHz running under the operating system Windows 7.

### Example 4.1

We consider the nonlinear fractional *q*-differential equation

59 $$ {}^{c}\mathcal{D}_{q}^{ \frac{11}{4}}[k](t) - \exp (t) - \frac{1}{2\sqrt{3}} k(t) \sin ^{2} t - \frac{1}{5}(1 - t)^{3} {} \mathcal{ D}_{q}^{\frac{3}{7}} [k](t) =0 $$

under the boundary conditions $k(0)=k''(0)=0$ and $k'(\frac{1}{5}) = \frac{2}{13} k''(1)$ for $t\in (0,1)$. It is clear that $\sigma = \frac{11}{4} \in (2,3)$, $\zeta = \frac{3}{7} \in (0,1)$, $r= \frac{1}{5} \in (0,1)$, and $\lambda = \frac{2}{13}>0$. We define the function $w: \overline{J}\times \mathbb{R}^{2} \to \mathbb{R}$ by

$$ w \bigl(t, k(t),l(t) \bigr)= \exp (t)+ \frac{1}{2\sqrt{3}} k(t) \sin ^{2} t + \frac{1}{5}(1 - t)^{3} l(t). $$

Let $k_{1}$, $k_{2}$, $l_{1}, l_{2} \in \mathbb{R}$. Then we have

$$\begin{aligned} & \bigl\vert w (t, k_{1},l_{1}) - w (t, k_{2}, l_{2}) \bigr\vert \\ &\quad = \biggl\vert \exp (t)+ \frac{1}{2\sqrt{3}} k_{1} \sin ^{2} t + \frac{1}{5}(1 - t)^{3} l_{1} - \biggl(\exp (t)+ \frac{1}{2\sqrt{3}} k_{2} \sin ^{2} t + (1 - t)^{3} l_{2} \biggr) \biggr\vert \\ & \quad \leq \frac{1}{\sqrt{3}} \vert k_{1} - k_{2} \vert \sin ^{2} t + \frac{1}{5} (1 - t)^{3} \vert l_{1} - l_{2} \vert . \end{aligned}$$

**Figure 1:**
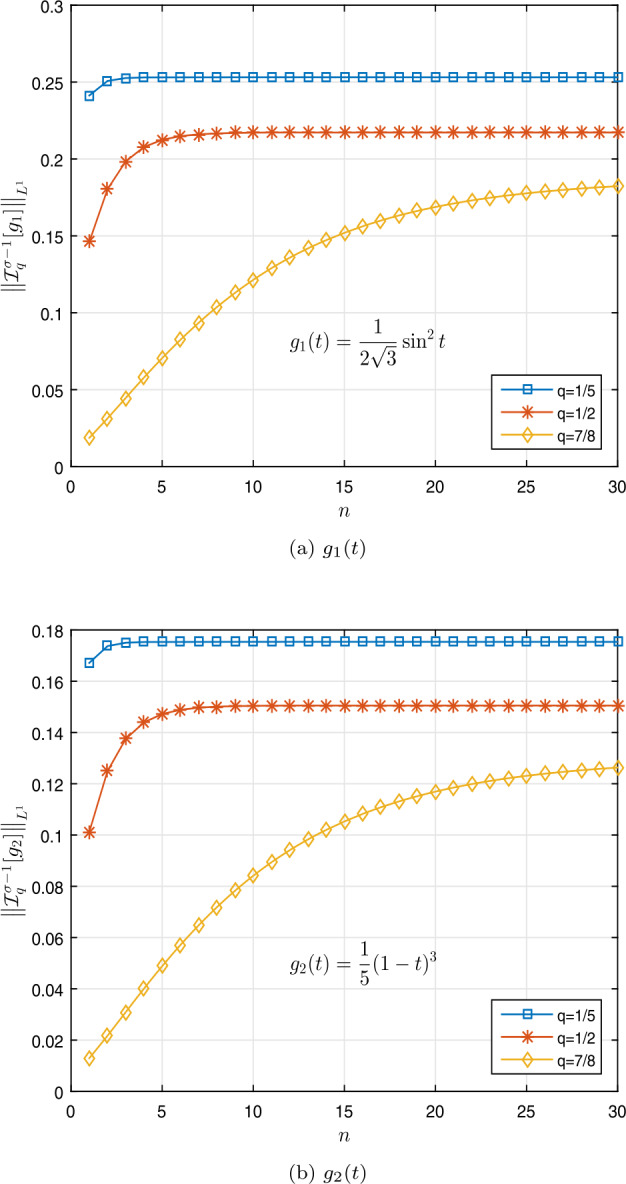
Graphical representation of $\Vert \mathcal{I}_{q}^{ \sigma -1} [g_{i}] \Vert _{L^{1}}$ for $q=\frac{1}{5}$, $\frac{1}{2}$, $\frac{7}{8}$ in Example 4.1

**Figure 2:**
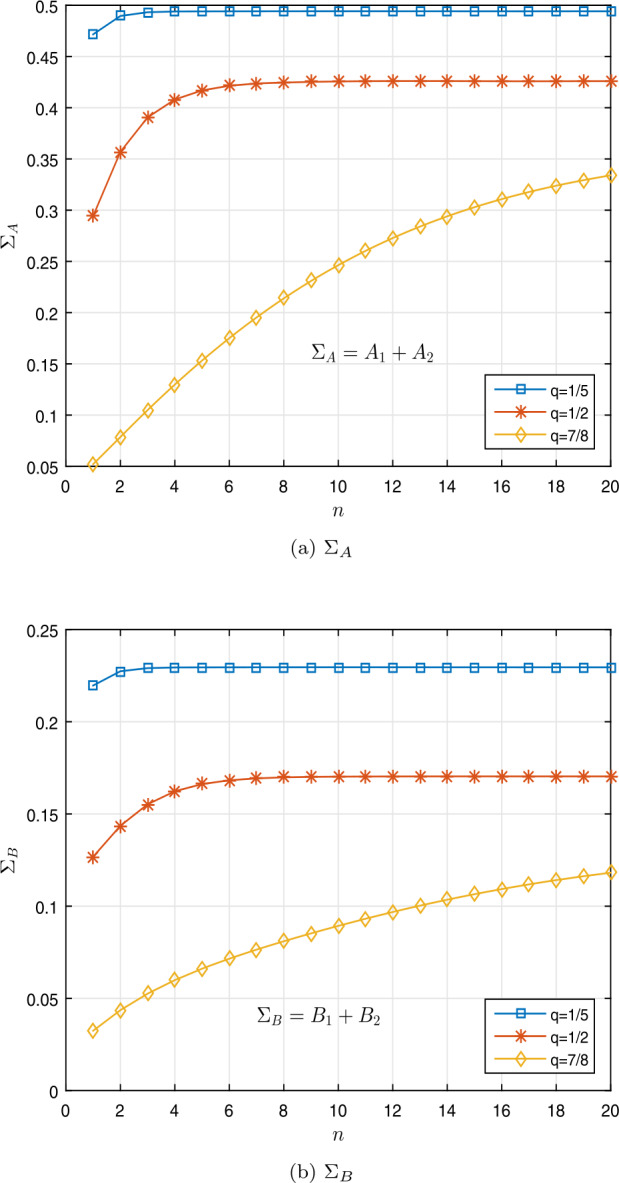
Graphical representation of $\Sigma _{A}$ and $\Sigma _{B}$ for $q =\frac{1}{5}$, $\frac{1}{2}$, $\frac{7}{8}$ in Example 4.1

Therefore $g_{1}(t) = \frac{1}{2\sqrt{3}}\sin ^{2} t$ and $g_{2}(t) = \frac{1}{5}(1-t)^{3}$, and by using equality (2) we obtain

$$\begin{aligned} &\mathcal{I}_{q}^{ \sigma -1} [g_{1}] (1)\approx 0.1402, 0.0770, 0.0378, \\ &\mathcal{I}_{q}^{ \sigma -1} [g_{2}] (1) \approx 0.0237, 0.0351, 1.2390 \end{aligned}$$

for $q=\frac{1}{5}$, $\frac{1}{2}$, $\frac{7}{8}$, respectively,

$$\begin{aligned} &\bigl\Vert \mathcal{I}_{q}^{ \sigma -1} [g_{1}] \bigr\Vert _{L^{1}} \approx 0.2531, 0.2172, 0.1872, \\ &\bigl\Vert \mathcal{I}_{q}^{ \sigma -1} [g_{2}] \bigr\Vert _{L^{1}} \approx 0.1754, 0.1505, 0.1297 \end{aligned}$$

for $q=\frac{1}{5}$, $\frac{1}{2}$, $\frac{7}{8}$, respectively, and

$$\begin{aligned} \Sigma _{A} & \approx 0.4941, 0.4259, 0.3687, \end{aligned}$$

which are less than one, for $q=\frac{1}{5}$, $\frac{1}{2}$, $\frac{7}{8}$, respectively,

$$\begin{aligned} & \Sigma _{B} \approx 0.2295< 0.7280= \biggl( \frac{4}{7} \biggr) \Gamma _{q} \biggl( \frac{4}{7} \biggr), \\ & \Sigma _{B}\approx 0.1704 < 0.8077 = \biggl( \frac{4}{7} \biggr) \Gamma _{q} \biggl( \frac{4}{7} \biggr), \\ & \Sigma _{B} \approx 0.1332 < 0.8728= \biggl( \frac{4}{7} \biggr) \Gamma _{q} \biggl( \frac{4}{7} \biggr) \end{aligned}$$

for $q=\frac{1}{5}$, $\frac{1}{2}$, $\frac{7}{8}$, respectively. Table 1 shows these results. Figures 2a and 2b show the curves of $\Sigma _{A}$ and $\Sigma _{B}$. Also, Figs. 1a and 1b show the curves of $\Vert \mathcal{I}_{q}^{ \sigma -1} [g_{1}] \Vert _{L^{1}}$ and $\Vert \mathcal{I}_{q}^{ \sigma -1} [g_{2}] \Vert _{L^{1}}$, respectively. Thus Theorem 3.3 implies that the nonlinear fractional *q*-differential equation (59) has a unique solution in $\mathfrak{B}$.

**Table 1:** Numerical results of problem (59) for $q = \frac{1}{5}$, $\frac{1}{2}$, $\frac{7}{8}$ and (1) $\Vert \mathcal{I}_{q}^{ \sigma -1} [g_{1}] \Vert _{L^{1}}$ and (2) $\Vert \mathcal{I}_{q}^{ \sigma -1} [g_{2}] \Vert _{L^{1}}$ in Example 4.1

*n*	$g_{1}(t)$			$g_{2}(t)$			$\Sigma _{A}$	$\Sigma _{B}$	$\frac{\Gamma _{q}(1- \zeta )}{(1-\zeta )^{-1}}$
	(1)	$A_{1}$	$B_{1}$	(2)	$A_{2}$	$B_{2}$			
	$q = \frac{ 1}{ 5}$								
1	0.2413	0.2941	0.1930	0.1672	0.2516	0.0267	0.4716	0.2196	0.7280
2	0.2507	0.3036	0.1930	0.1737	0.2630	0.0344	0.4896	0.2274	0.7280
3	0.2526	0.3055	0.1930	0.1750	0.2653	0.0361	0.4932	0.2291	0.7280
4	0.2530	0.3058	0.1930	0.1753	0.2658	0.0364	0.4939	0.2294	0.7280
5	0.2531	0.3059	0.1930	0.1753	0.2659	0.0365	0.4941	0.2295	0.7280
6	0.2531	0.3059	0.1930	0.1753	0.2659	0.0365	0.4941	0.2295	0.7280
7	0.2531	0.3059	0.1930	0.1754	0.2659	0.0365	0.4941	0.2295	0.7280
8	0.2531	0.3059	0.1930	0.1754	0.2659	0.0365	0.4941	0.2295	0.7280
9	0.2531	0.3059	0.1930	0.1754	0.2659	0.0365	0.4941	0.2295	0.7280
	$q = \frac{ 1}{2}$								
1	0.1461	0.1876	0.1161	0.1012	0.1067	0.0107	0.2943	0.1268	0.8077
2	0.1804	0.2225	0.1188	0.1250	0.1345	0.0248	0.3569	0.1436	0.8077
3	0.1985	0.2406	0.1192	0.1375	0.1499	0.0361	0.3906	0.1552	0.8077
⋮	⋮	⋮	⋮	⋮	⋮	⋮	⋮	⋮	⋮
9	0.2169	0.2591	0.1192	0.1503	0.1663	0.0509	0.4254	0.1701	0.8077
10	0.2171	0.2593	0.1192	0.1504	0.1664	0.0510	0.4257	0.1702	0.8077
11	0.2171	0.2593	0.1192	0.1504	0.1665	0.0511	0.4258	0.1703	0.8077
12	0.2172	0.2594	0.1192	0.1505	0.1665	0.0511	0.4259	0.1703	0.8077
13	0.2172	0.2594	0.1192	0.1505	0.1665	0.0511	0.4259	0.1704	0.8077
14	0.2172	0.2594	0.1192	0.1505	0.1665	0.0511	0.4259	0.1704	0.8077
15	0.2172	0.2594	0.1192	0.1505	0.1665	0.0511	0.4259	0.1704	0.8077
	$q = \frac{ 7}{8}$								
1	0.0187	0.0387	0.0320	0.0129	0.0134	0.0004	0.0521	0.0324	0.4938
2	0.0313	0.0560	0.0426	0.0217	0.0226	0.0010	0.0785	0.0436	0.5608
3	0.0446	0.0722	0.0507	0.0309	0.0323	0.0018	0.1045	0.0526	0.6119
⋮	⋮	⋮	⋮	⋮	⋮	⋮	⋮	⋮	⋮
21	0.1710	0.2046	0.0713	0.1185	0.1338	0.0485	0.3383	0.1199	0.8535
22	0.1730	0.2065	0.0714	0.1199	0.1355	0.0501	0.3421	0.1214	0.8560
23	0.1748	0.2083	0.0714	0.1211	0.1371	0.0514	0.3454	0.1228	0.8581
24	0.1763	0.2098	0.0714	0.1222	0.1384	0.0527	0.3483	0.1240	0.8600
⋮	⋮	⋮	⋮	⋮	⋮	⋮	⋮	⋮	⋮
50	0.1869	0.2204	0.0714	0.1295	0.1478	0.0616	0.3682	0.1330	0.8725
51	0.1870	0.2205	0.0714	0.1295	0.1478	0.0617	0.3683	0.1330	0.8725
52	0.1870	0.2205	0.0714	0.1296	0.1479	0.0617	0.3684	0.1331	0.8726
53	0.1870	0.2206	0.0714	0.1296	0.1479	0.0617	0.3685	0.1331	0.8726
54	0.1871	0.2206	0.0714	0.1296	0.1479	0.0618	0.3685	0.1331	0.8726
55	0.1871	0.2206	0.0714	0.1296	0.1480	0.0618	0.3686	0.1331	0.8727
56	0.1871	0.2206	0.0714	0.1296	0.1480	0.0618	0.3686	0.1332	0.8727
57	0.1871	0.2206	0.0714	0.1296	0.1480	0.0618	0.3686	0.1332	0.8727
58	0.1871	0.2207	0.0714	0.1297	0.1480	0.0618	0.3687	0.1332	0.8727
59	0.1872	0.2207	0.0714	0.1297	0.1480	0.0618	0.3687	0.1332	0.8728
60	0.1872	0.2207	0.0714	0.1297	0.1480	0.0619	0.3687	0.1332	0.8728

### Example 4.2

In this example, we apply Theorem 3.4 to prove that the fractional *q*-differential equation

60 $$ {}^{c}\mathcal{D}_{q}^{\frac{8}{3}} [k] (t) = \biggl(1 - \frac{1}{t+1} \biggr)^{2} \biggl[ \frac{(k(t))^{2} }{ 6 + (k(t))^{4} } + \ln \bigl( 1 + \bigl({}^{c} \mathcal{D}_{q}^{\frac{4}{5}} [k] (t) \bigr)^{2} \bigr) +1 \biggr], $$

under the boundary conditions $k(0)=k''(0)=0$ and $k' ( \frac{1}{4} ) = \frac{5}{3} k''(1)$ for $t\in (0,1)$, has at least one nontrivial solution. It is obvious that $\sigma = \frac{8}{3} \in (2,3)$, $\zeta = \frac{6}{11} \in (0,1)$, $r= \frac{1}{4} \in (0,1)$, and $\lambda = \frac{5}{3}>0$. We define function the $w: \overline{J} \times \mathbb{R}^{2} \to \mathbb{R}$ by

$$\begin{aligned} w \bigl(t, k(t),l(t) \bigr)& = \biggl(1 - \frac{1}{t+1} \biggr)^{2} \biggl[ \frac{(k(t))^{2} }{ 6 + (k(t))^{4} } + \ln \bigl( 1 + \bigl(l(t) \bigr)^{2} \bigr) +1 \biggr]. \end{aligned}$$

**Figure 3:**
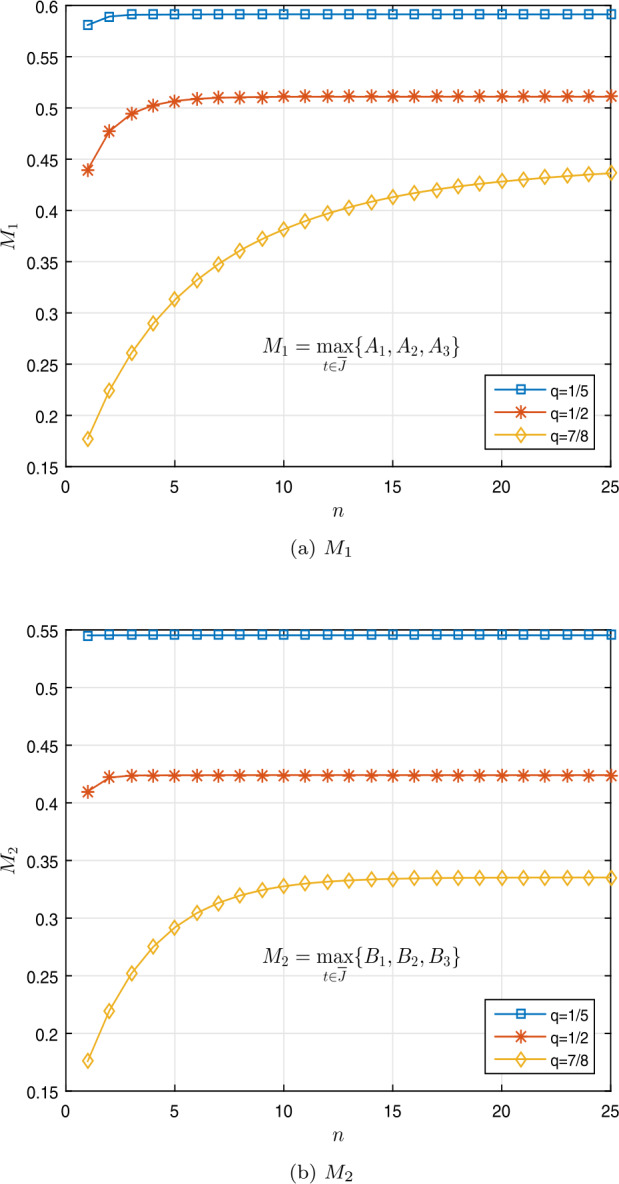
Graphical representation of $M_{1}$ and $M_{2}$ for $q = \frac{1}{5}$, $\frac{1}{2}$, $\frac{7}{8}$ in Example 4.2

Figures 3a and 3b show the curves of $M_{1}$ and $M_{2}$. Let *k*, $\overline{k} \in \mathbb{R}$. Then we have

$$\begin{aligned} \begin{aligned} \bigl\vert w ( t, k, \overline{k} ) \bigr\vert & = \biggl( 1 - \frac{1}{t+1} \biggr)^{2} \biggl\vert \frac{(k(t))^{2} }{ 6 + (k(t))^{4} } + \ln \bigl( 1 + \bigl( \overline{k}(t) \bigr)^{2} \bigr) +1 \biggr\vert \\ & \leq \biggl( 1 - \frac{1}{t+1} \biggr)^{2} \frac{(k(t))^{2} }{ 6 + (k(t))^{4} } + \biggl( 1 - \frac{1}{t+1} \biggr)^{2} \ln \bigl( 1 + \bigl( \overline{k}(t) \bigr)^{2} \bigr) + \biggl( 1 - \frac{1}{t+1} \biggr)^{2}. \end{aligned} \end{aligned}$$

Now from inequality (26) we can consider $g_{i}(t)= ( 1 - \frac{1}{t+1} )^{2}$ for $i=1,2, 3$ and

$$\begin{aligned} &\phi _{1} \bigl( \bigl\vert k(t) \bigr\vert \bigr) = \frac{(k(t))^{2} }{ 6 + (k(t))^{4} }, \\ &\phi _{2} \bigl( \bigl\vert \overline{k}(t) \bigr\vert \bigr) = \ln \bigl( 1 + \bigl( \overline{k}(t) \bigr)^{2} \bigr). \end{aligned}$$

Let us find *η* such that inequality (27) holds. In this case, by (14) we calculate $A_{i}$ and $B_{i}$ for $i=1,2,3$. We obtain

$$\begin{aligned} & A_{1} \approx 0.5913, 0.5109, 0.4445, \qquad B_{1} \approx 0.5453, 0.4240, 0.3353, \\ &A_{2} \approx 0.5913, 0.5109, 0.4445, \qquad B_{2} \approx 0.5453, 0.4240, 0.3353, \\ &A_{3} \approx 0.5913, 0.5109, 0.4445, \qquad B_{3} \approx 0.5453, 0.4240, 0.3353, \end{aligned}$$

for $q=\frac{1}{5}$, $\frac{1}{2}$, $\frac{7}{8}$, respectively, and so

$$\begin{aligned} & p \biggl(q=\frac{1}{5} \biggr) = M_{1} + \frac{ M_{2}}{ (1-\zeta ) \Gamma _{\frac{1}{5}}( 1- \zeta )}\approx 1.5075, \\ & p \biggl(q=\frac{1}{2} \biggr)= M_{1} + \frac{ M_{2}}{ (1-\zeta ) \Gamma _{\frac{1}{2}}( 1- \zeta )}\approx 1.1474, \\ &p \biggl(q=\frac{7}{8} \biggr) = M_{1} + \frac{ M_{2}}{ (1-\zeta ) \Gamma _{\frac{7}{8}}( 1- \zeta )}\approx 0.9073. \end{aligned}$$

**Figure 4:**
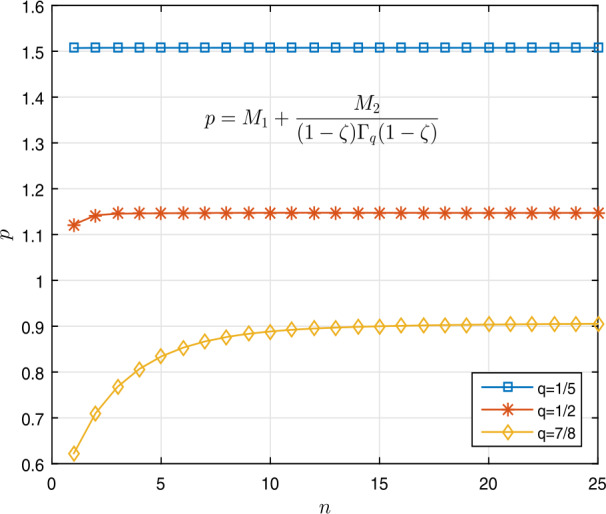
2D graphs of $p= M_{1} + \frac{ M_{2}}{ (1-\zeta ) \Gamma _{\frac{1}{5}}( 1- \zeta )}$ for $q = \frac{1}{5}$, $\frac{1}{2}$, $\frac{7}{8}$ in Example 4.2

Tables 2 and 3 show these results. Also, Fig. 4 shows the curve of the *p* base on Table 2 for $q=\frac{1}{5}, \frac{1}{2}, \frac{7}{8}$. Now we see that inequality (27) is equivalent to

61 $$\begin{aligned} & \bigl[ \phi _{1}(\eta ) + \phi _{2}(\eta ) + 1 \bigr] p -\eta = \biggl[ \frac{\eta ^{2} }{ 6 + \eta ^{4} } + \ln \bigl( 1 + \eta ^{2} \bigr) +1 \biggr](1.5075) - \eta < 0, \\ &\bigl[ \phi _{1}(\eta ) + \phi _{2}(\eta ) + 1 \bigr] p -\eta = \biggl[ \frac{\eta ^{2} }{ 6 + \eta ^{4} } + \ln \bigl( 1 + \eta ^{2} \bigr) +1 \biggr](1.1474) - \eta < 0, \\ &\bigl[ \phi _{1}(\eta ) + \phi _{2}(\eta ) + 1 \bigr] p -\eta = \biggl[ \frac{\eta ^{2} }{ 6 + \eta ^{4} } + \ln \bigl( 1 + \eta ^{2} \bigr) +1 \biggr](0.9073) - \eta < 0, \end{aligned}$$

for $q=\frac{1}{5}$, $\frac{1}{2}$, $\frac{7}{8}$, respectively. Now by using Algorithm 6 we try to find a suitable value for *η* in inequalities (61). The algorithm is created for the same problems. On the other hand, the results show that it works exactly. According to Table 4, the suitable values of *η* in (61) are $\eta =4, 5, 8$ for $q =\frac{1}{5}$, $\frac{1}{2}$, $\frac{7}{8}$, respectively. Note that $\Omega (\eta )$ defined by

$$ \Omega (\eta )= \bigl[ \phi _{1}(\eta ) + \phi _{2}( \eta ) +1 \bigr] \biggl( M_{1} + \frac{M_{2}}{(1 - \zeta ) \Gamma _{q} (1 - \zeta )} \biggr) -\eta $$

is negative for values of *η*. Thus Theorem 3.4 implies that the nonlinear fractional *q*-differential equation (60) has at least one nontrivial solution in $\mathfrak{B}$.

**Algorithm 6:**
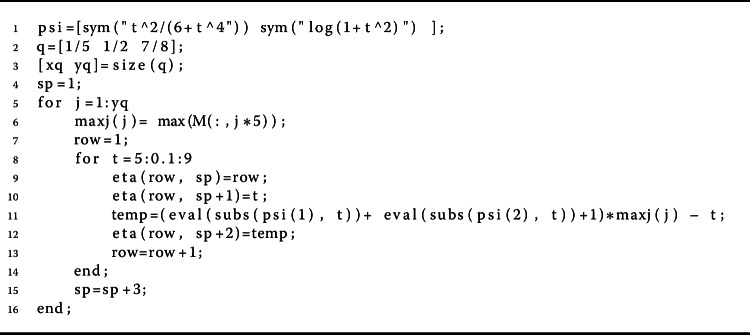
MATLAB lines for finding suitable values of *η* in Eq. (27) for *q* variable in Example 4.2

**Table 2:** Numerical results of problem (59) for $q = \frac{1}{5}$, $\frac{1}{2}$, $\frac{7}{8}$ and (1) $\Vert \mathcal{I}_{q}^{ \sigma -1} [g_{1}] \Vert _{L^{1}}$, (2) $\Vert \mathcal{I}_{q}^{ \sigma -1} [g_{2}] \Vert _{L^{1}}$, and (3) $\Vert \mathcal{I}_{q}^{ \sigma -1} [g_{3}] \Vert _{L^{1}}$ in Example 4.2

*n*	(1)	$A_{1}$	$B_{1}$	(2)	$A_{2}$	$B_{2}$	(3)	$A_{3}$	$B_{3}$
	$g_{1}(t)$			$g_{2}(t)$			$g_{3}(t)$		
	$q = \frac{ 1}{ 5}$								
1	0.2125	0.5809	0.5452	0.2125	0.5809	0.5452	0.2125	0.5809	0.5452
2	0.2207	0.5892	0.5453	0.2207	0.5892	0.5453	0.2207	0.5892	0.5453
3	0.2224	0.5909	0.5453	0.2224	0.5909	0.5453	0.2224	0.5909	0.5453
4	0.2227	0.5912	0.5453	0.2227	0.5912	0.5453	0.2227	0.5912	0.5453
5	0.2228	0.5913	0.5453	0.2228	0.5913	0.5453	0.2228	0.5913	0.5453
6	0.2228	0.5913	0.5453	0.2228	0.5913	0.5453	0.2228	0.5913	0.5453
7	0.2228	0.5913	0.5453	0.2228	0.5913	0.5453	0.2228	0.5913	0.5453
	$q = \frac{ 1}{2}$								
1	0.1327	0.4399	0.4099	0.1327	0.4399	0.4099	0.1327	0.4399	0.4099
2	0.1630	0.4773	0.4219	0.1630	0.4773	0.4219	0.1630	0.4773	0.4219
3	0.1789	0.4943	0.4237	0.1789	0.4943	0.4237	0.1789	0.4943	0.4237
4	0.1871	0.5026	0.4240	0.1871	0.5026	0.4240	0.1871	0.5026	0.4240
5	0.1912	0.5068	0.4240	0.1912	0.5068	0.4240	0.1912	0.5068	0.4240
⋮	⋮	⋮	⋮	⋮	⋮	⋮	⋮	⋮	⋮
10	0.1953	0.5108	0.4240	0.1953	0.5108	0.4240	0.1953	0.5108	0.4240
11	0.1953	0.5109	0.4240	0.1953	0.5109	0.4240	0.1953	0.5109	0.4240
12	0.1954	0.5109	0.4240	0.1954	0.5109	0.4240	0.1954	0.5109	0.4240
13	0.1954	0.5109	0.4240	0.1954	0.5109	0.4240	0.1954	0.5109	0.4240
14	0.1954	0.5109	0.4240	0.1954	0.5109	0.4240	0.1954	0.5109	0.4240
	$q = \frac{ 7}{8}$								
1	0.0187	0.1773	0.1759	0.0187	0.1773	0.1759	0.0187	0.1773	0.1759
2	0.0309	0.2244	0.2199	0.0309	0.2244	0.2199	0.0309	0.2244	0.2199
3	0.0435	0.2607	0.2518	0.0435	0.2607	0.2518	0.0435	0.2607	0.2518
⋮	⋮	⋮	⋮	⋮	⋮	⋮	⋮	⋮	⋮
24	0.1626	0.4350	0.3352	0.1626	0.4350	0.3352	0.1626	0.4350	0.3352
25	0.1638	0.4362	0.3352	0.1638	0.4362	0.3352	0.1638	0.4362	0.3352
26	0.1648	0.4373	0.3353	0.1648	0.4373	0.3353	0.1648	0.4373	0.3353
27	0.1658	0.4382	0.3353	0.1658	0.4382	0.3353	0.1658	0.4382	0.3353
28	0.1666	0.4390	0.3353	0.1666	0.4390	0.3353	0.1666	0.4390	0.3353
⋮	⋮	⋮	⋮	⋮	⋮	⋮	⋮	⋮	⋮
50	0.1719	0.4444	0.3353	0.1719	0.4444	0.3353	0.1719	0.4444	0.3353
51	0.1720	0.4444	0.3353	0.1720	0.4444	0.3353	0.1720	0.4444	0.3353
52	0.1720	0.4444	0.3353	0.1720	0.4444	0.3353	0.1720	0.4444	0.3353
53	0.1720	0.4445	0.3353	0.1720	0.4445	0.3353	0.1720	0.4445	0.3353
54	0.1721	0.4445	0.3353	0.1721	0.4445	0.3353	0.1721	0.4445	0.3353
55	0.1721	0.4445	0.3353	0.1721	0.4445	0.3353	0.1721	0.4445	0.3353
56	0.1721	0.4445	0.3353	0.1721	0.4445	0.3353	0.1721	0.4445	0.3353

**Table 3:** Numerical results of $M_{1}$, $M_{2}$, and $p= M_{1}+ \frac{M_{2}}{(1-\zeta )\Gamma _{q}(1- \zeta )} $ for $q = \frac{1}{5}$, $\frac{1}{2}$, $\frac{7}{8}$ in Example 4.2

*n*	$q = \frac{ 1}{ 5}$			$q = \frac{ 1}{ 2}$			$q = \frac{7}{8}$		
	$M_{1}$	$M_{2}$	*p*	$M_{1}$	$M_{2}$	*p*	$M_{1}$	$M_{2}$	*p*
1	0.5809	0.5452	1.5070	0.4399	0.4099	1.1219	0.1773	0.1759	0.6224
2	0.5892	0.5453	1.5075	0.4773	0.4219	1.1421	0.2244	0.2199	0.7104
3	0.5909	0.5453	1.5075	0.4943	0.4237	1.1455	0.2607	0.2518	0.7677
4	0.5912	0.5453	1.5075	0.5026	0.4240	1.1465	0.2894	0.2750	0.8067
⋮	⋮	⋮	⋮	⋮	⋮	⋮	⋮	⋮	⋮
9	0.5913	0.5453	1.5075	0.5107	0.4240	1.1473	0.3722	0.3243	0.8834
10	0.5913	0.5453	1.5075	0.5108	0.4240	1.1473	0.3817	0.3276	0.8884
11	0.5913	0.5453	1.5075	0.5109	0.4240	1.1474	0.3899	0.3299	0.8921
12	0.5913	0.5453	1.5075	0.5109	0.4240	1.1474	0.3969	0.3316	0.8949
13	0.5913	0.5453	1.5075	0.5109	0.4240	1.1474	0.4029	0.3327	0.8970
⋮	⋮	⋮	⋮	⋮	⋮	⋮	⋮	⋮	⋮
40	0.5913	0.5453	1.5075	0.5109	0.4240	1.1474	0.4435	0.3353	0.9071
41	0.5913	0.5453	1.5075	0.5109	0.4240	1.1474	0.4437	0.3353	0.9072
42	0.5913	0.5453	1.5075	0.5109	0.4240	1.1474	0.4438	0.3353	0.9072
43	0.5913	0.5453	1.5075	0.5109	0.4240	1.1474	0.4439	0.3353	0.9072
44	0.5913	0.5453	1.5075	0.5109	0.4240	1.1474	0.4440	0.3353	0.9072
45	0.5913	0.5453	1.5075	0.5109	0.4240	1.1474	0.4441	0.3353	0.9073
46	0.5913	0.5453	1.5075	0.5109	0.4240	1.1474	0.4442	0.3353	0.9073
47	0.5913	0.5453	1.5075	0.5109	0.4240	1.1474	0.4442	0.3353	0.9073

**Table 4:** Numerical results for finding suitable values of *η* in equation (27) for $q = \frac{1}{5}$, $\frac{1}{2}$, $\frac{7}{8}$ in Example 4.2, where $\Omega (\eta )= [ \phi _{1}(\eta ) + \phi _{2}(\eta ) +1 ] ( M_{1} + \frac{M_{2}}{(1 - \zeta ) \Gamma _{q} (1 - \zeta )} ) -\eta $

*n*	*η*	Ω(*η*)<0		
		$q = \frac{1}{ 5}$	$q = \frac{ 1}{ 2}$	$q = \frac{7}{8}$
1	3.0000	2.1347	0.9079	0.0906
2	3.1000	2.1154	0.8693	0.0392
3	3.2000	2.0943	0.8294	
4	3.3000	2.0715	0.7882	−0.0668
⋮	⋮	⋮	⋮	⋮
9	3.8000	1.9351	0.5649	−0.3480
10	3.9000	1.9036	0.5170	−0.4068
11	4.0000	1.8708	0.4681	−0.4663
12	4.1000	1.8367	0.4183	−0.5266
13	4.2000	1.8014	0.3676	−0.5877
⋮	⋮	⋮	⋮	⋮
18	4.7000	1.6077	0.1007	−0.9033
19	4.8000	1.5658	0.0449	−0.9684
20	4.9000	1.5229		−1.0340
21	5.0000	1.4790	−0.0690	−1.1002
22	5.1000	1.4342	−0.1270	−1.1670
23	5.2000	1.3884	−0.1857	−1.2344
⋮	⋮	⋮	⋮	⋮
47	7.6000	0.0745	−1.7591	−2.9806
48	7.7000	0.0126	−1.8301	−3.0577
49	7.8000		−1.9015	−3.1351
50	7.9000	−0.1126	−1.9732	−3.2127
51	8.0000	−0.1759	−2.0452	−3.2906
52	8.1000	−0.2395	−2.1176	−3.3687
53	8.2000	−0.3037	−2.1903	−3.4471

### Example 4.3

In this example, we consider the fractional *q*-differential equation

62 $$\begin{aligned} {}^{c}\mathcal{D}_{q}^{\frac{18}{7}} [k](t) & = \frac{1-t^{2}}{1 + t^{2}} \biggl[ \frac{3\pi }{k(t) + {}^{c}\mathcal{D}_{q}^{\frac{5}{6}} [k](t) + 6\pi } + \exp \bigl( -\pi \bigl(k(t) + {}^{c}\mathcal{D}_{q}^{ \frac{5}{6}} [k](t) \bigr) \bigr) \biggr] \end{aligned}$$

under boundary conditions $k(0) = k''(0) =0$ and $k' ( \frac{3}{8} ) = \frac{15}{4} k''(1)$ for $t\in (0,1)$ such that the assumptions of Lemma 3.6 hold. Clearly, $\sigma = \frac{18}{7} \in (2,3)$, $\zeta = \frac{5}{6} \in (0,1)$, $r= \frac{3}{8} \in (0,1)$, and $\lambda = \frac{15}{4}>0$. Also, $\lambda (\sigma -2)= \frac{15}{7} >1$. Table 5 shows that$\Lambda \approx 1.1887$, 1.0505, 0.9579 for $q = \frac{1}{5}$, $\frac{1}{2}$, $\frac{7}{8}$, respectively, which we calculated by Algorithm 7. In the algorithm, we define the matrix for saving the results for $q=\frac{1}{5}, \frac{1}{2}, \frac{7}{8}$. We define the function $w: \overline{J}\times \mathbb{R}^{2} \to \mathbb{R}$ by

$$\begin{aligned} w \bigl(t, k(t),l(t) \bigr) & = \frac{1-t^{2}}{1 + t^{2}} \biggl[ \frac{3\pi }{k(t) + l(t) + 6\pi } + \exp \bigl( -\pi \bigl(k(t) + l(t) \bigr) \bigr) \biggr]. \end{aligned}$$

**Figure 5:**
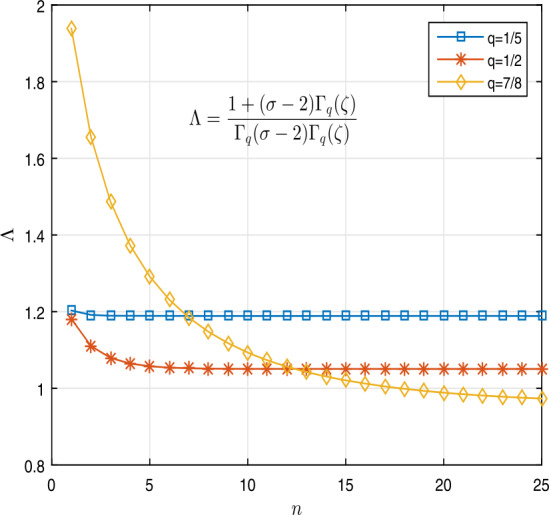
2D graphs of $\Lambda =\frac{1+ (\sigma -2) \Gamma _{q}( \zeta ) }{\Gamma _{q}( \sigma -2) \Gamma _{q}(\zeta ) }$ for $q = \frac{1}{5}$, $\frac{1}{2}$, $\frac{7}{8}$ in Example 4.3

Figure 5 shows the curve of the Λ base on Table 5 for $q=\frac{1}{5}, \frac{1}{2}, \frac{7}{8}$. If we define the functions $\gamma : \mathbb{R}^{2} \to \mathbb{R}$ and $\mu : \overline{J} \to \mathbb{R}^{+}$ by

$$ \gamma \bigl(k(t),l(t) \bigr) = \frac{3\pi }{k(t) + l(t) + 6\pi } + \exp \bigl( -\pi \bigl(k(t) + l(t) \bigr) \bigr) $$

and $\mu (t) = \frac{1-t^{2}}{1 + t^{2}} $, then assumption (A1) holds. Now we verify assumption (A2). Let

$$\begin{aligned} \varrho _{q}(\xi ) & = \textstyle\begin{cases} \frac{15}{4},& \xi > \frac{3}{8}, \\ \frac{15}{4} - \frac{ ( \frac{3}{8} - q\xi )^{ ( \frac{18}{7} - 2 )} (1 - q\xi )^{ (\frac{18}{7}-3 ) }}{ \frac{18}{7} - 2}, & \xi \leq \frac{3}{8}, \end{cases}\displaystyle \\ & = \textstyle\begin{cases} \frac{15}{4},& \xi > \frac{3}{8}, \\ \frac{15}{4} - \frac{7}{4} ( \frac{3}{8} - q\xi )^{ (\frac{4}{7} ) } ( 1 - q\xi )^{ (-\frac{3}{7} )}, & \xi \leq \frac{3}{8}. \end{cases}\displaystyle \end{aligned}$$

Therefore

63 $$\begin{aligned} & \Gamma _{q} \biggl(\frac{4}{7} \biggr) \mathcal{I}_{q}^{\frac{4}{7}} \bigl[ \mu ( \xi ) \varrho _{q}(\xi ) \bigr](1) \\ &\quad = \int _{0}^{1} (1- q \xi )^{ (\frac{18}{7}-3 )} \mu ( \xi ) \varrho _{q}(\xi ) \,\mathrm{d}_{q}\xi \\ &\quad = \int _{0}^{1} ( 1 - q \xi )^{ ( \frac{4}{ 7} )} \frac{ 1 - \xi ^{2}}{1 + \xi ^{2}} \varrho _{q}(\xi ) \,\mathrm{d}_{q} \xi \\ &\quad = \textstyle\begin{cases} 0.36485,& q=\frac{1}{5}, \\ 0.68838,& q=\frac{1}{2}, \\ 0.63316,& q=\frac{7}{8}. \end{cases}\displaystyle \end{aligned}$$

**Figure 6:**
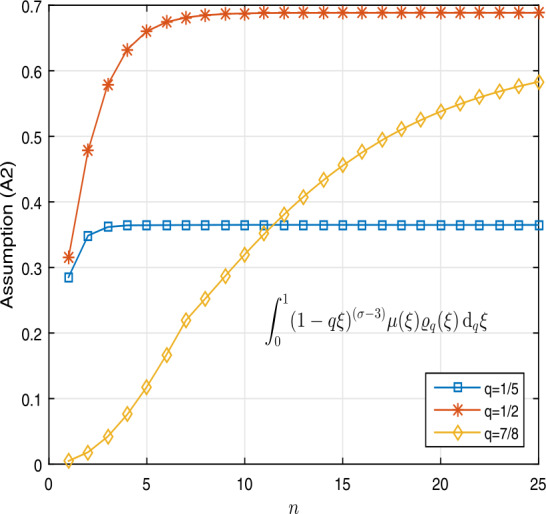
2D graphs of $\int _{0}^{1} (1- q \xi )^{ (\sigma -3)} \mu ( \xi ) \varrho _{q}( \xi ) \,\mathrm{d}_{q}\xi $ for $q = \frac{1}{5}$, $\frac{1}{2}$, $\frac{7}{8}$ in Example 4.3

So assumption (A2) holds. Table 6 shows these results. For this, we use Algorithm 8. Figure 6 shows the results of equation (63). On the other hand,

$$\begin{aligned} &\begin{aligned} L_{0} & = \lim_{( \vert k \vert + \vert l \vert ) \to 0} \frac{\gamma (k, l)}{ \vert k \vert + \vert l \vert }, \\ & = \lim_{( \vert k \vert + \vert l \vert ) \to 0} \frac{1}{ \vert k \vert + \vert l \vert } \biggl[ \frac{3\pi }{k(t) + l(t) + 6\pi } + \exp \bigl( -\pi \bigl(k(t) + l(t) \bigr) \bigr) \biggr] \\ & = \infty , \end{aligned} \\ &\begin{aligned} L_{\infty }& = \lim_{( \vert k \vert + \vert l \vert ) \to \infty } \frac{\gamma (k, l)}{ \vert k \vert + \vert l \vert } \\ & = \lim_{( \vert k \vert + \vert l \vert ) \to \infty } \frac{1}{ \vert k \vert + \vert l \vert } \biggl[ \frac{3\pi }{k(t) + l(t) + 6\pi } + \exp \bigl( -\pi \bigl(k(t) + l(t) \bigr) \bigr) \biggr] \\ & = 0. \end{aligned} \end{aligned}$$

Thus by Theorem 3.7 we get that problem (62) has at least one nonnegative solution.

**Algorithm 7:**
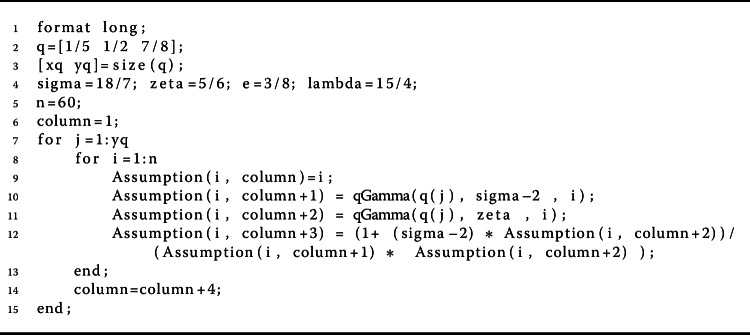
MATLAB lines for calculating values of $\Lambda = \frac{1+ (\sigma -2) \Gamma _{q}(\zeta ) }{\Gamma _{q}(\sigma -2) \Gamma _{q}(\zeta ) }$ in Theorem 3.7 for *q* variable in Example 4.3

**Algorithm 8:**
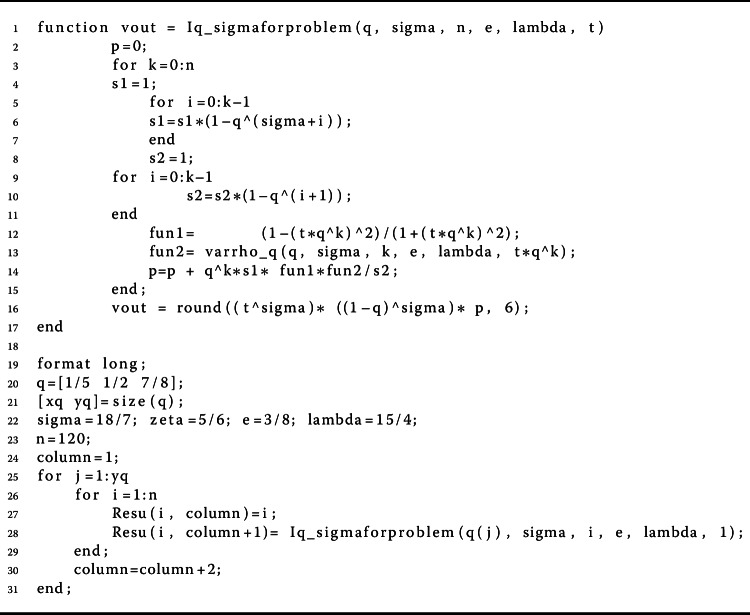
MATLAB lines for calculating $\int _{0}^{1} (1- q \xi )^{ (\sigma -3)} \mu (\xi ) \varrho _{q}(\xi ) \,\mathrm{d}_{q}\xi $ in Assumption (A2) for *q* variable in Example 4.3

**Table 5:** Numerical results of $\Gamma _{q}(\sigma -2)$, $\Gamma _{q}(\zeta )$, and $\Lambda = \frac{1+ (\sigma -2) \Gamma _{q}( \zeta ) }{\Gamma _{q}( \sigma -2) \Gamma _{q}(\zeta ) }$ in equation (62) for $q = \frac{1}{5}$, $\frac{1}{2}$, $\frac{7}{8}$ in Example 4.3

*n*	$q = \frac{ 1}{ 5}$			$q = \frac{ 1}{ 2}$			$q = \frac{7}{8}$		
	$\Gamma _{q}(\sigma -2)$	$\Gamma _{q}(\zeta )$	Λ	$\Gamma _{q}(\sigma -2)$	$\Gamma _{q}(\zeta )$	Λ	$\Gamma _{q}(\sigma -2)$	$\Gamma _{q}(\zeta )$	Λ
1	1.2612	1.0573	1.2030	1.2839	1.0584	1.1809	0.8642	0.9059	1.9386
2	1.2714	1.0600	1.1915	1.3507	1.0773	1.1103	0.9815	0.9493	1.6555
3	1.2734	1.0605	1.1892	1.3826	1.0861	1.0792	1.0708	0.9805	1.4860
4	1.2738	1.0606	1.1888	1.3982	1.0905	1.0646	1.1416	1.0043	1.3727
5	1.2739	1.0606		1.4059	1.0926	1.0575	1.1991	1.0231	1.2917
6	1.2739	1.0606	1.1887	1.4097	1.0936	1.0540	1.2466	1.0382	1.2311
7	1.2739	1.0606	1.1887	1.4116	1.0942	1.0522	1.2863	1.0506	1.1843
8	1.2739	1.0606	1.1887	1.4126	1.0944	1.0514	1.3197	1.0608	1.1473
9	1.2739	1.0606	1.1887	1.4131	1.0945	1.0509	1.3480	1.0694	1.1176
10	1.2739	1.0606	1.1887	1.4133	1.0946	1.0507	1.3722	1.0767	1.0933
11	1.2739	1.0606	1.1887	1.4134	1.0946	1.0506	1.3929	1.0828	1.0733
12	1.2739	1.0606	1.1887	1.4135	1.0947		1.4106	1.0881	1.0566
13	1.2739	1.0606	1.1887	1.4135	1.0947	1.0505	1.4260	1.0925	1.0426
14	1.2739	1.0606	1.1887	1.4135	1.0947	1.0505	1.4392	1.0964	1.0308
15	1.2739	1.0606	1.1887	1.4136	1.0947	1.0505	1.4506	1.0997	1.0208
16	1.2739	1.0606	1.1887	1.4136	1.0947	1.0505	1.4605	1.1026	1.0122
⋮	⋮	⋮	⋮	⋮	⋮	⋮	⋮	⋮	⋮
51	1.2739	1.0606	1.1887	1.4136	1.0947	1.0505	1.5269	1.1214	0.9582
52	1.2739	1.0606	1.1887	1.4136	1.0947	1.0505	1.5270	1.1215	0.9582
53	1.2739	1.0606	1.1887	1.4136	1.0947	1.0505	1.5271	1.1215	0.9581
54	1.2739	1.0606	1.1887	1.4136	1.0947	1.0505	1.5271	1.1215	0.9581
55	1.2739	1.0606	1.1887	1.4136	1.0947	1.0505	1.5272	1.1215	0.9580
56	1.2739	1.0606	1.1887	1.4136	1.0947	1.0505	1.5272	1.1215	0.9580
57	1.2739	1.0606	1.1887	1.4136	1.0947	1.0505	1.5273	1.1215	0.9580
58	1.2739	1.0606	1.1887	1.4136	1.0947	1.0505	1.5273	1.1215	
59	1.2739	1.0606	1.1887	1.4136	1.0947	1.0505	1.5273	1.1216	0.9579
60	1.2739	1.0606	1.1887	1.4136	1.0947	1.0505	1.5273	1.1216	0.9579

**Table 6:** Numerical results of $\int _{0}^{1} (1- q \xi )^{ (\sigma -3)} \mu ( \xi ) \varrho _{q}( \xi ) \,\mathrm{d}_{q}\xi $ for $\sigma =\frac{18}{7}$ in Assumption (A2) and for $q = \frac{1}{5}$, $\frac{1}{2}$, $\frac{7}{8}$ in Example 4.3

*n*	$0<\int _{0}^{1} (1- q \xi )^{ (\sigma -3)} \mu ( \xi ) \varrho _{q}(\xi ) \,\mathrm{d}_{q}\xi < 1$		
	$q = \frac{ 1}{ 5}$	$q = \frac{ 1}{ 2}$	$q = \frac{7}{8}$
1	0.28515	0.31485	0.00482
2	0.34871	0.47839	0.01824
3	0.36161	0.57866	0.04195
4	0.36420	0.63251	0.07572
5	0.36472	0.66022	0.11804
6	0.36482	0.67425	0.16674
7	0.36484	0.68130	0.21946
8	0.36485	0.68484	0.25289
9	0.36485	0.68661	0.28682
10	0.36485	0.68750	0.31984
11	0.36485	0.68794	0.35125
⋮	⋮	⋮	⋮
15	0.36485	0.68836	0.45528
16	0.36485	0.68837	0.47565
17	0.36485	0.68838	0.49394
18	0.36485	0.68838	0.51030
19	0.36485	0.68838	0.52486
⋮	⋮	⋮	⋮
77	0.36485	0.68838	0.63313
78	0.36485	0.68838	0.63314
79	0.36485	0.68838	0.63314
80	0.36485	0.68838	0.63315
81	0.36485	0.68838	0.63315
82	0.36485	0.68838	0.63315
83	0.36485	0.68838	0.63316
84	0.36485	0.68838	0.63316
85	0.36485	0.68838	0.63316

## Conclusion

The *q*-differential boundary equations and their applications represent a matter of high interest in the area of fractional *q*-calculus and its applications in various areas of science and technology. *q*-differential boundary value problems occur in the mathematical modeling of a variety of physical operations. In the end of this paper, we investigated a complicated case by utilizing an appropriate basic theory. An interesting feature of the proposed method is replacing the classical derivative with *q*-derivative to prove the existence of nonnegative solutions for a familiar problem for *q*-differential equations on a time scale, and under suitable assumptions, we have presented the global convergence of the proposed method with the line searches. The results of numerical experiments demonstrated the effectiveness of the proposed algorithm.

## Data Availability

Data sharing not applicable to this paper as no datasets were generated or analyzed during the current study.
